# Client, health care provider, and policymaker perspectives on point-of-care/near-point-of-care testing for sexually transmitted infections in community pharmacies in Uganda

**DOI:** 10.1371/journal.pone.0347364

**Published:** 2026-10-15

**Authors:** Annet A. Onzia, Adelline Twimukye, Johan H. Melendez, Matthew M. Hamill, Peter Kyambadde, Agnes Kiragga, Yukari C. Manabe, Rosalind Parkes-Ratanshi

**Affiliations:** 1 Infectious Diseases Institute, Makerere University, Kampala, Uganda; 2 Johns Hopkins University School of Medicine, Baltimore, Maryland, United States of America; 3 Ministry of Health of Uganda, National STI Control Unit, Kampala, Uganda, Uganda; 4 African Population and Health Research Center, Kitisuru, Nairobi, Kenya; 5 Centre for Public Health, Queen’s University Belfast, Belfast, Northern Ireland, United Kingdom; GGD Amsterdam, NETHERLANDS, KINGDOM OF THE

## Abstract

**Introduction:**

Point-of-care or near-point-of-care (POC/near-POC) tests for sexually transmitted infections (STIs) are often unavailable and unaffordable in low-resource settings, despite the World Health Organization’s (WHO) recommendation that such testing be offered, leading to empiric treatment. Private community pharmacies (CPs) are often the first point of contact for people with symptoms of STIs, where pharmacists dispense treatment without diagnostic testing or prescriptions. This study evaluated clients,’ providers,’ and policymakers’ perspectives on POC/near-POC STI testing in community pharmacies for targeted management.

**Methods:**

Between June and September 2022, we nested a qualitative study into a prospective cohort of clients seeking both STI and non-STI treatments in community pharmacies in Kampala and Wakiso districts in Uganda. They were tested for HIV, syphilis, trichomonas, chlamydia, and gonorrhea using POC/near-POC tests. A purposive sample of 50 participants consented to participate in in-depth and key informant interviews. Data were analyzed thematically using NVivo version 14 with an inductive approach, with results reported according to the Consolidated Criteria for Reporting Qualitative Research (COREQ).

**Results:**

Clients (n = 35), health care providers (n = 9), and policymakers (n = 6) described the benefits of POC/near-POC tests for HIV/STIs at community pharmacies, citing affordability, accessibility, and convenience. Participants noted that such testing could promote behavioral change, shorten turnaround time for results, speed access to treatment relative to conventional laboratory methods, and support sustainable antimicrobial resistance control. Reported barriers included limited awareness among clients and health workers, inadequate privacy and space, long waiting times, unclear self-sample collection instructions, stigma around HIV testing, and reluctance to test for non-HIV STIs. Participants recommended raising STI awareness, offering clearer explanations of test results, expanding test access, reducing stigma, provider training, and ensuring a sustainable supply chain for testing kits.

**Conclusions:**

POC/near-POC testing for HIV/STIs was highly acceptable to both pharmacy clients and providers in this study. Integrating such testing into community pharmacy settings could strengthen national HIV/STI management programs, and addressing the identified barriers could improve access to HIV/STI diagnostics and strengthen linkage to care.

## Introduction

Sub-Saharan Africa (SSA) bears a disproportionate share of the global sexually transmitted infections (STI) burden, with most research centered on HIV, while non-HIV STIs remain under-researched in the region [1,2]. STIs may cause serious adverse health outcomes, including pelvic inflammatory disease, infertility, congenital infections, and neonatal death. Non-HIV STIs increase the risk of HIV transmission and continue to fuel the ongoing HIV epidemic in the region [3].The current syndromic approach to STI management misses asymptomatic cases, leads to continued transmission, and may result in overtreatment, contributing to antimicrobial resistance (AMR) [4]. To address the rising global STI prevalence, there is an urgent need to shift to an etiologic approach [4]. Point-of-care or near-point-of-care (POC/near-POC) tests offer greater flexibility compared to conventional laboratory methods and can be used in rural and community settings, providing quick results that aid faster decision-making and improve care efficiency [5,6]. Lateral flow assays are widely accepted in low- and middle-income countries (LMICs) [7–9] and are the backbone of HIV and syphilis diagnostic testing in these settings.

In Uganda, poor access to STI services in public healthcare has led many clients to seek care in private and informal sectors, such as community pharmacies and traditional healers [10]. Community pharmacies, often called retail pharmacies, are private-for-profit drug outlets, where pharmacists or nurse dispensers sell prescribed and compounded medications. They may offer a convenient and discreet setting for STI testing and treatment, with more flexible hours that often include round-the-clock availability. In addition, many provide broader public health services, including antiretroviral treatment (ART) and sexual and reproductive health (SRH) care [11]. As trusted health care providers, pharmacy staff offer private consultations in settings already involved in sexual health, including condom sales and emergency contraception [11,12]. Whilst there are about 3595 licensed pharmacies in Uganda that comply with Ministry of Health guidelines [13], there are many unlicensed premises where prescription-only antibiotics for STI treatment are often available over the counter, leading to an increased risk of incorrect or inadequate treatment, which contributes to AMR [14–16]. Whilst some community pharmacies have been approved by the Ministry of Health to provide ART, many operate in isolation with limited integration into the national health care system [17,18]. Whereas data from high-income countries show the feasibility and acceptability of STI/HIV testing in community pharmacies [19], there are limited data on its implementation in LMICs like Uganda.

Among individuals seeking medication and other services at community pharmacies in Kampala and Wakiso districts, Uganda, we conducted a prospective cohort study to evaluate the feasibility and uptake of POC/near-POC STI testing as an alternative to traditional facility-based testing services [20]. The findings showed that pharmacy-based POC/near-POC STI testing is both feasible and acceptable, with good participation from clients. This study also revealed a significant burden of STIs, factors linked to service use, and various implementation challenges. To gain a deeper understanding of the observed quantitative results, we carried out a nested qualitative study that explored clients’, providers’, and policymakers’ views on awareness, acceptability, and satisfaction with POC/near-POC STI/HIV testing in community pharmacies. This is essential for evaluating the potential for consistent, high-quality services [21] identifying training needs and exploring opportunities for integration into the national HIV/STI management policy.

## Methods

### Study design

We conducted a nested qualitative study from June 7, 2022, to September 13, 2022, with a purposive sample of 50 participants, employing in-depth and key informant interviews. These participants were enrolled in a prospective cohort study (S1 File) conducted from May 2021 to November 2023 among 450 participants seeking both STI and non-STI treatment in community pharmacies [20]. In brief, participants were screened and enrolled by trained nurse counsellors stationed within the participating pharmacies. These nurse counsellors collected venous blood samples for POC HIV and syphilis testing using the SD Bioline (Abbott, Illinois, US), while participants self-collected genital specimens (vaginal swabs for women, penile-meatal swabs for symptomatic males, or urine for asymptomatic males) for chlamydia, gonorrhoea, and trichomoniasis testing, guided by pictorial illustrations in a private or semi-private area of the pharmacy or nearby sanitary facilities. TV samples were processed on-site using POC OSOM^®^ Trichomonas Rapid Test (Sekisui, San Diego, USA) platforms by the nurse counsellors, whereas CT/NG samples were transported to a nearby laboratory for testing using the GeneXpert system (Cepheid, HBDC, Maurens-Scopont, France), a near-POC test. Study staff interpreted all results and disclosed them jointly to the pharmacist and participant to ensure shared understanding and acceptance of the diagnosis. All study participants received free sexual health education, including counselling and STI/HIV treatment. All participants who tested positive for any curable STI were treated by the study team (not by pharmacy staff independently) upon dissemination of test results, in line with national treatment guidelines [22]. Partner notification for those with positive tests for STIs included in-person or through phone calls or through an interactive voice response tool (Call for Life) [23]. All participants were followed up and re-tested for all STIs, except HIV, on days 30 and 90 to ascertain treatment response, symptom resolution following the baseline visit (when applicable), and partner notification for people who tested positive for any STI at previous visits. In this manuscript, POC testing refers to the HIV, syphilis, and TV testing that were performed and resulted within the premises of the community pharmacy, while NG and CT tests are considered near-POC tests, as their samples were transferred to the main laboratory and results received between 12 and 24 hours later.

### Study settings

The study was conducted in 18 private community pharmacies located in Kampala city and the neighboring peri-urban district of Wakiso in Uganda, with a population of approximately 3.8 and 3.5 million people, respectively [24]. By March 2020, there were approximately 353 licensed pharmacies in the five divisions of Kampala and about 66 in Wakiso District [24].

### Sampling size estimation

Using maximum variation sampling, we purposively selected 54 participants for in-depth interviews (IDIs) and key informant interviews (KIIs) across five divisions of Kampala and municipalities in Wakiso District, Uganda. Of these, four declined to participate due to time constraints. We conducted 35 in-depth interviews with clients purposively selected from among those enrolled in the main study at community pharmacies. Participants were originally recruited into the main study under the following categories: those seeking emergency contraception, STI treatment, and those seeking care for other unrelated conditions. Both male and female participants were included, and purposive selection continued until data saturation was achieved.

We also conducted 15 key informant interviews with health care providers and stakeholders, including pharmacists, dispensers, representatives from the Ministry of Health National AIDS Control Program, the Pharmaceutical Society of Uganda, and the National Drug Authority. Key informants were selected for their expertise in STI management and/or pharmacy operations. Interviews took place either at the participating community pharmacies or at the Infectious Diseases Institute clinic to ensure privacy.

### Ethical considerations

The study was approved by the Institutional Review Board (IRB) at The Joint Clinical Research Center (JC1820) in Uganda and the Johns Hopkins University (IRB00279241). The Uganda National Council of Science and Technology (HS1274ES) provided overall regulatory and administrative oversight and approval regarding the conduct of study activities. We obtained further permission from the district health offices of the two districts in Kampala and Wakiso, as well as from the proprietors of the individual pharmacies where the study activities took place. Written informed consent was obtained from all participants before enrollment. For participants deemed illiterate or semi-illiterate, we used a thumbprint in the presence of an impartial witness. Participation in the study was entirely voluntary, and whoever wished to withdraw was allowed to do so without any repercussions. All pharmacy staff signed a confidentiality agreement with the study to ensure the confidentiality and privacy of study participants enrolled from their facilities. All study participants received time compensation of ~$10 USD, as guided by the IRB. All participants’ personal information and identifiers were removed and anonymized during analysis and reporting using alphanumeric codes to ensure confidentiality. All data were password-protected and stored on encrypted computers and locked cabinets.

### Data collection

Data were collected by study team members experienced in qualitative data collection in both English and Luganda (the predominant local language). Both in-depth interviews and key informant interviews used semi-structured guides (S2 File and S3 File) in participants’ preferred language, lasting 30–60 minutes. Interviews were audio-recorded and supplemented with written notes. The topic guides, based on Sekhon et al.’s acceptability framework [25] and the Ugandan Ministry of Health's STI Management Guidelines [22], covered areas such as knowledge, perceived benefits and risks, barriers, and attitudes towards POC/near-POC STI/HIV testing in community pharmacies. Data collection was staggered, allowing emerging results from in-depth interviews to be verified during subsequent key informant interviews and continuing until saturation was reached.

### Data analysis

The audio data were translated into English, if recorded in another language, transcribed, coded, and thematically analyzed to address the study objectives. To ensure transferability, we used a thick description of the findings [26]. Data accuracy and completeness were verified by an independent review of all transcripts. Analysis was conducted using NVivo version 14 software and a thematic approach. A flexible coding framework (S4 File) was developed to facilitate inter-district comparisons and accommodate emerging themes. Each transcript was read multiple times, and relevant text segments were coded. Similar codes were grouped to form themes, which helped develop narratives. Contradictory data were treated as potential different viewpoints and analyzed further to validate them. Validated data enriched insights on the research topic. To ensure rigor, each transcript was independently coded, compared, and discussed by four study team members. Emerging themes were reviewed and agreed upon by the study team. In this manuscript, we report key phrases and verbatim quotes from study participants’ experiences and perspectives according to the Consolidated Criteria for Reporting Qualitative Research (COREQ) [27].

## Results

Among 35 participants in the in-depth interviews, the median age was 29 (IQR 25–40) years, with the majority female, 57.1% (n = 20), married 51.4% (n = 18); 14.3% (n = 5) had post-secondary education (**Table 1).** The reasons for visiting the pharmacy were as follows: 54.3% (n = 19) sought care for STI symptoms, 8.6% (n = 3) wanted emergency contraception, and 37.1% (n = 13) had visited for other reasons besides the two.

**Table 1:** Participant characteristics for the in-depth interviews.

Variable	Frequency (%), n = 35
**Sex**	
Female	20 (57.1)
Male	15 (42.9)
**Age** (Median (IQR))	29 (25-40)
**Highest level of education**	
Informal/none	1 (2.9)
Primary	14 (40.0)
Secondary	15 (42.9)
Tertiary	3 (8.6)
University	2 (5.7)
**Marital status**	
Married	18 (51.4)
Separated	6 (17.1)
Single	11 (31.4)
**Reason for Visiting the Pharmacy**	
Other reason	13 (37.1)
Emergency Contraception	3 (8.6)
Symptomatic of STI	19 (54.3)
**Occupation category**	
Formal employment	3 (8.6)
Informal employment	28 (80.0)
Student	2 (5.7)
Unemployed	2 (5.7)

*One participant visited the pharmacy for both STI symptoms and emergency contraception and was considered under the STI symptoms category*

For the 15 key informant interviews, the median age was 39 (IQR 28–43) years, 60% (n = 9) were male, 40% (n = 6) were female, and the majority, 66.5% (n = 10), had post-diploma education (Table 2).

**Table 2:** Characteristics of participants for the key informants’ interviews.

Variable	Frequency (%), n = 15
**Sex**	
Female	6 (40.0)
Male	9 (60.0)
**Age** (Median (IQR))	39 (IQR 28–43)
**Highest level of education**	
Certificate	2 (13.3)
Diploma	3 (20.0)
Bachelor’s degree	2 (13.3)
Master's (post-graduate degree)	7 (46.7)
PhD	1 (6.7)
**Position in the community/occupation**	
Health care providers	9 (60)
Policy makers	6 (40)

The analysis identified four main themes related to POC/near-POC testing for STIs/HIV at community pharmacies. The first theme revealed participants’ general perceptions about the benefits of POC/near-POC testing, including the quick turnaround time, quality of services, and counseling services offered. The second theme assessed the impact of POC/near-POC testing for STIs/HIV at community pharmacies. The third emerging theme identified the social and structural barriers to POC/near-POC STI/HIV testing at community pharmacies, while the fourth theme identified recommendations by participants for the implementation of POC/near-POC STI/HIV testing at community pharmacies. These themes cut across various levels at the individual, interpersonal, health systems, and policy levels.

### Theme 1: Benefits of services offered through the main study at community pharmacies

#### Good quality and comprehensive services offered.

Our study participants highlighted the good quality and comprehensive nature of the services, which included counselling, education on HIV/STI prevention and treatment, medication provision, and follow-up calls. The health education and counselling, which focused on prevention, testing, and treatment of STIs/HIV, emphasized the importance of knowing one’s health status and making informed decisions, as emphasized by one of the participants who was diagnosed with HIV.

“… *I felt scared a bit, but then since I received good counselling, I said let me enroll for treatment so that I can practice positive living*...*But when she [nurse] calls me…I get encouraged, and I conclude that, when I am on treatment, I can get well”*-- IDI, Male, symptomatic.

#### Reduced costs of POC/near-POC testing.

Participants appreciated the availability of free POC/near-POC testing offered by the main study, contrasted to the high costs typically associated with conventional STI testing. This made testing more accessible, especially for those who previously lacked resources or were deterred by the high costs of consultation and laboratory fees in routine practice. However, the extent to which the acceptability and uptake observed here would be sustained under a fee-for-service model remains unclear.

“*Their services were good, and yet they were free*. *Exorbitant costs are usually associated with traditional STI testing…. consultation before you get in*…*then lab fees*…” --IDI, Female, asymptomatic.

#### Ease of accessibility.

Community pharmacies were viewed as convenient, being well-positioned and accessible in both urban and rural settings. They were seen as trusted spaces for testing, with clients appreciating their proximity and the regulatory standards that ensured quality.

“*Pharmacies are now very many…I no longer waste time to go to hospitals since they will tell me there are no drugs there and send me to buy at a pharmacy*.” -- IDI, Female, Symptomatic

“*Community pharmacies are very accessible…there is some kind of trust between the community and the pharmacy*. *They are quite well positioned… to penetrate deeper areas. Go to any upcoming town, and you will find a pharmacy there.*” --KII, MOH technical advisor for vaccines and therapeutics.

#### Ease of use of POC/near-POC tests.

POC/near-POC tests were considered convenient and easy to use, with some health care workers feeling empowered to perform tests for pharmacy clients within the pharmacy, which contributed to faster diagnoses and treatment management.

*“...we can just do it [test] ourselves, find out the truth, then we manage”*-KII, Pharmacy dispenser.

### Theme 2: Impact of the POC/near-POC testing for STIs at community pharmacies

#### Sexual health education.

Participants reported that the education they received with POC/near-POC testing encouraged changes in sexual behavior. Testing encouraged clients to adopt what they described as safer sexual practices, including partner reduction, mutual disclosure of status, and encouraging partners to also get tested.

“...*My biggest lesson has been that it is always important to test and know your HIV status because it will impact your overall sexual relations…after this testing, I chose to stick to my officially introduced wife, and I openly told her, I am done with [other] women, let me settle down with you and we raise our children” --* IDI, Male, asymptomatic.

“*I am not supposed to have many sexual partners. You need to have one partner and encourage him to be checked like you.”-* IDI, Female, symptomatic.

#### Etiological diagnosis for targeted management.

Participants noted that POC/near-POC testing led to accurate diagnoses and targeted treatment, which could result in recovery from curable STIs and improved health outcomes, compared to the standard of care syndromic management for STIs available at other facilities. Participants acknowledged the significance of rapid testing before treatment to ensure the appropriate medications were prescribed.

“*In the context of antimicrobial resistance, this could go miles in reducing the time someone accesses effective treatment, so I see them to be the future.”* --KII, Male, MOH Consultant

#### Quick results turnaround time.

The quick turnaround time of POC/near-POC tests was seen as a major advantage, particularly for those with limited time who might not visit public health facilities. The turnaround time of results made it easier for clients to begin treatment promptly. This also led to encouragement for their sexual partners to get tested if found positive for STIs/HIV, implying that they wanted to avoid reinfection by encouraging their partners to get tested and treated for STIs/HIV following POC/near-POC testing.

*“It saves a lot of time. Someone will be out in 15 minutes…they will tell you that I have no time to go to the lab. I am too busy. But if they come to you and you tell them, just give me 10 minutes. I carry out the test; they are [so much] willing.” --*KII, Pharmacy dispenser.

*“I took it upon myself to inform my partner to get tested after me…the tests are easy and quick.” --*IDI, Male, Symptomatic

### Theme 3: Barriers to STI testing in community pharmacies

#### Lack of awareness about POC/near-POC tests among clients and providers.

Both clients and health workers often lacked awareness of POC/near-POC testing for STIs apart from HIV, since the usual practice was to describe symptoms to pharmacists and receive medication without being tested. Most participants had attended the community pharmacies to buy medicine to treat various non-STI symptoms. Some health workers recognized the need to educate both the public and practitioners about the accuracy and benefits of POC/near-POC testing, which could increase its uptake if available.

“*No, I had never heard about it. I just bumped into it*.” --IDI, Male, Symptomatic

*“There is a knowledge gap…if the public could know that point of care is accurate…quick, then they would take it up…that should start with medical workers” --* KII, male, pharmacist.

#### Sample collection challenges.

Many participants noted that community pharmacies lacked privacy, as tests were conducted and results provided in open spaces, discouraging honest communication and increasing fear. There was insufficient space and/or a designated bathroom to conduct tests and engage clients comfortably, which meant that participants used public or other shop toilets.

“...*the pharmacy was in the open, and people kept passing…I was a bit scared …it did not have privacy.” --* IDI, Female, symptomatic.

“*Yes, because you have to go into people’s toilets…You might find that it is locked…at the trading center, you had to first pay to use the toilet*.” --IDI, Female, Symptomatic

Some male clients found the instructions for self-sample collection unclear, leading to discomfort and possible inaccuracies in sample collection. The process of collecting samples was difficult for some participants, despite clear instructions given, which deterred many from testing.

“*At some point, I didn’t know how deep I was to push the swab…She [nurse] didn’t tell me how far I can put it”* --IDI, Female, symptomatic.

#### Inadequate training of pharmacy providers.

Pharmacists expressed concerns regarding the lack of training around POC/near-POC testing, further questioning the quality of the testing process and the validity of results.

“*Do we have the expertise to collect the right sample? In the hospital, you have a lab tech…I don’t think, as a pharmacist, we have sufficient training; our training tells us so much…how to collect a sample…simple things like a prick, an HIV test that is okay”* --KII, Female, pharmacist.

#### Long queues and waiting times.

Our participants observed that community pharmacies are often busy, with long queues that discouraged testing and made waiting for results more difficult. In some cases, additional confirmatory testing (especially for HIV), was required, which added to clients’ anxiety and deterred some people from testing altogether. Despite eventually receiving negative results, one participant described significant anxiety while waiting in the queue for her turn to be tested and to receive her results.

“*The worry about standing in long queues for long in the open deters people… although I had negative results for all the STIs…I wondered when she is [nurse] calling me, why is she not?”* --IDI, Female, symptomatic.

#### Stigma associated with HIV testing.

The stigma associated with HIV testing, particularly the fear of receiving positive results, was a significant barrier to POC/near-POC testing. Some participants were afraid of the emotional and social consequences of knowing their HIV status, such as isolation or self-harm in an environment with no counsellors*.*

“*Some people are afraid to know their status. Some others might take poison if they find out that they have HIV.”* --KII, Male, pharmacy technician.

#### Concerning the diagnostic performance of POC/near-POC tests.

Health workers raised concerns about the accuracy and reliability of POC/near-POC tests, particularly regarding false positives and negatives

“*The challenge with POC/near-POCs is sensitivity and specificity… the false negatives and false positives.” --*KII, Male, Consultant medical officer

#### Potential risks of antimicrobial resistance.

Participants expressed concern that placing POC/near-POC test kits in pharmacies could lead to misuse, as pharmacists may lack the clinical training needed to use them properly, potentially contributing to antimicrobial resistance.

“*Why not put these kits and support the clinics which are designated to do real patient care…other than equipping the drug outlets which are run by a little bit lower cadre … the placement of POC/near-POC test kits in pharmacies is likely to perpetuate antimicrobial resistance (AMR) as it encourages pharmacists to undertake clinical work for which they have inadequate training*.” --KII, MOH STI control program.

The profit-driven nature of pharmacies was seen as a potential conflict of interest, where financial incentives might take precedence over patient care, leading to inappropriate tests and/or prescriptions.

“*It happens a lot in pharmacies... dispensers are under pressure, overlooking health to focus on money”*-IDI, Female, asymptomatic.

### Theme 4: Recommendations for implementing POC/near-POC Testing at community pharmacies

#### Raising awareness of POC/near-POC testing and reducing stigma.

Participants recommended that more sensitization about the availability of POC/near-POC testing for HIV and STIs is needed using contextually relevant methods. This sensitization needs to be matched with counselling to reduce stigma around STI/HIV testing, addressing fears, shame, and misinformation during community sensitization campaigns.

“*It would be good if things like flyers are put there…Messages that say testing is for free on this and that day…. the use of loudspeakers going through communities encouraging people to get information about that sexually transmitted disease, signs and symptoms.”* --IDI, Female, symptomatic.

Participants recommended that additional public health sensitization around antimicrobial resistance and misuse of antibiotics in both humans and animals is urgently required.

“*We may need to create messages that are easy to understand, for the local population, so they know that it is not good to go and buy antibiotics without a prescription for themselves or their animals*… *Drugs have been misused not only in human beings but in animals. So, the emergence of resistance is the biggest challenge around, misuse of drugs, under doses*.” --KII, medical officer.

#### Contextually appropriate POC/near-POC testing.

To increase access to POC/near-POC testing, participants recommended that testing points be accessible and convenient within local communities, for example, with dedicated testing days to reduce queues and waiting times.

“*I think they should get some days in a week and say these are the days they test*.” –IDI, Female, Asymptomatic

#### Address privacy concerns at community pharmacies.

To enhance privacy, participants recommended creating safe spaces within or outside community pharmacies to facilitate open, confidential communication between clients and health practitioners, further encouraging POC/near-POC testing.

“*We get like a tent, that is placed on the side, so that the client and the health worker…you are just in your conversation.”*—IDI, Female, Symptomatic

*“I think point of care is a cheap option compared to the conventional laboratory one…It also saves a lot of space, as I have said, I could create some small room there that basically runs point of care.”—*KII, Male, Pharmacist

#### Use of alternative non-invasive samples for testing.

Participants further recommended that easy-to-collect samples, like urine, saliva, or small blood drops, be used to increase uptake of POC/near-POC testing at community pharmacies.

“...*it has to be samples that are culturally and technologically friendly, like saliva, urine.” --*KII, Male, STI Control Unit

Further, participants recommended making the POC/near-POC tests easier to use by translating instructions into local languages to enable self-sample collection and results interpretation, as asserted by one of the participants.

“...*let them even try to bring it in Luganda [local language] to make it easy for someone to read it”* --KII, Female, comprehensive nurse/dispenser.

#### POC/near-POC testing to support antimicrobial stewardship.

The use of POC/near-POC tests to support antimicrobial stewardship was recognized and welcomed by providers and policymakers. Participants suggested that the government strengthen the enforcement of regulations to prevent the over-the-counter sale of STI treatments by non-registered pharmacies. Supervision could help enforce laws to prohibit the sale of antibiotics for STIs without proper testing and prescription, as this contributes significantly to AMR. Others emphasized the need for pharmacists to refer clients to qualified clinicians for appropriate treatment and for engagement with traditional healers.

“*No one goes to check at my pharmacy to see if the prescriptions dispensed and antibiotics brought in…no regulation is the major stumbling block.”* –KII, Male, Pharmacist

*“Even the herbalists have contributed to AMR. They mix their herbs…get some doxy[doxycycline], get some streptomycin, and boil…So when someone says, “I can treat kabotongo[syphilis],” they are more than sure; they know what they put inside there…in that herbal, they have laced it with conventional medicine. Of course, it can’t be stable because they have boiled it, mixed it with other things…if we can involve the traditional medicines…like opinion leaders, traditional council…we can let them know about the dangers of mixing the herbals with the conventional medicine.” --*KII, Male, National Drug Authority

POC/near-POC tests were recognized as supporting a move from syndromic management of STIs to testing-based treatment with more control of AMR.

“*If the ministry found personnel to visit the community pharmacies to ensure drugs are dispensed on prescription...following up the prescription books…regularly, it will force dispensers…to tell clients to test before prescription.” --* KII, Male, Consultant medical officer.

#### Regular review and evidence-based update to local guidelines.

Updating policies to reflect current evidence and best practices to effectively address emerging challenges, especially around AMR, is important. Regular training on new policies was emphasized.

“*The guidelines should be informed by actual local AMR surveillance and not necessarily just changed because of literature adopted anywhere*.” –KII, Male, STI Control Unit

*“We should really have this constant training to update our knowledge”-*KII, Female, pharmacist.

#### Ensuring the constant availability of POC/near-POC tests.

Participants recommended that POC/near-POC testing kits and treatment need to be consistently available in community pharmacies for a sustainable and trustworthy service.

“...*the supply chain has to be guaranteed and sustainable*” --KII, Pharmacist, vaccines, drugs supply chain.

However, not all our participants welcomed POC/near-POC testing. Some challenged the concept of POC/near-POC testing, as they felt this should be done only in clinics.

“...*these pharmacies, their mandate…are not licensed to treat…so why not equip the nearby clinics and then they get drugs from the pharmacy...Why not put these kits and support the clinics…which are designated to do real patient care…other than equipping the drug outlets which are run by a little bit lower cadre?”* --KII, Medical officer, consultant.

## Discussion

In this study, POC/near-POC testing for HIV/STIs was broadly acceptable to clients, providers, and policymakers, who valued the affordability, accessibility, and convenience it offered compared to conventional laboratory-based testing. Participants described how faster turnaround times could support quicker treatment initiation, promote behavioral change, and contribute to more sustainable AMR control by moving away from empiric treatment. However, testing in this study was provided free of charge, and it remains unclear whether the same level of acceptability and uptake would hold if clients were required to pay out of pocket, which limits the generalizability of these findings to real-world settings. Moreover, several substantial barriers emerged alongside these benefits, most notably inadequate privacy and limited physical space within pharmacies, which participants identified as a significant deterrent to testing uptake, particularly for stigmatized conditions such as HIV. Additional challenges, including low awareness among clients and health workers, long waiting times, unclear self-sample collection instructions, and reluctance to test for STIs beyond HIV, further suggest that translating this acceptability into sustained, real-world use will require deliberate attention to structural and contextual constraints, not simply the introduction of testing technology itself.

Before the study, both clients and providers had limited awareness of POC/near-POC testing, mainly due to inadequate community sensitization, consistent with previous SSA research linking low awareness of HIV/STI POC/near-POC testing to low uptake of services [28]. Nonetheless, acceptability and satisfaction with POC/near-POC testing among users and health care providers was high. Participants valued the ease of sample collection, rapid result interpretation, and same-day treatment, which encouraged testing uptake, treatment completion, and partner notification, consistent with previous studies that POC/near-POC testing saves time and cost [7,29]. However, some health care providers expressed dissatisfaction with test instructions being available only in English, which limited client comprehension, findings consistent with a study in India where language barriers left users feeling disempowered to interpret diagnostic results [30].

Our study identified several barriers to POC/near-POC testing for STI/HIV testing at community pharmacies. The lack of privacy, which hindered open communication and likely discouraged uptake, was consistent with previous studies [6]. Participants proposed setting up tents or designated safe spaces to ensure confidentiality and privacy. Inadequate sanitary facilities for women who needed to collect vaginal samples meant many paid for nearby facilities, creating an inconvenience that discouraged testing. Self-sample collection, especially invasive methods, was compounded by these privacy concerns, with many recommending less invasive sample options, such as urine- or saliva-based tests, to improve comfort and encourage testing, findings consistent with prior research [31,32]. Notably, urine and saliva are not currently validated specimen types for HIV/syphilis testing, so this reflects a preference for improving acceptability rather than a proposed standard testing protocol change. Psychological barriers, mainly the fear of receiving an HIV diagnosis associated with stigma and shame, also deterred testing. This aligns with previous reports that pharmacy-based psychosocial support increased willingness to test, reduced anxiety [33] and improved engagement with follow-up care [34]. Participants also raised concerns about pharmacists’ training and potential conflicts between professional ethics and commercial interests, questioning pharmacists’ competence in independent sample collection and testing. Notably, in this study, all sample collection, testing, and treatment were performed by trained study nurses and laboratory staff rather than routine pharmacy personnel, required for both procedural consistency and to comply with Ugandan regulations restricting diagnostics to authorized laboratory personnel [35]. Our findings therefore speak to the acceptability of pharmacy-based testing, not to demonstrated capacity of pharmacy staff to perform POC/near-POC testing. Participants suggested revising guidelines to allow specialized certification for sample collection and management, while others argued that testing should remain the responsibility of clinicians and laboratory professionals to ensure diagnostic integrity. Similar regulatory debates have been reported in South Africa, where pilot programs demonstrated that structured training, certification, and quality assurance mechanisms could enable safe expansion of pharmacy-based HIV testing while maintaining diagnostic integrity [36]. Lastly, participants noted that rapid tests are widely available for HIV, whereas POC/near-POCTs for non-HIV STIs remain largely unavailable, consistent with global evidence of limited availability and varied field performance of non-HIV STI tests [4], constraining feasible integration into pharmacy-based care. Beyond pharmacy regulation, antimicrobial stewardship emerged as a broader challenge in this study. Key informants described traditional healers combining herbal preparations with antibiotics such as doxycycline and streptomycin, an unregulated practice with unpredictable dosing. Addressing this will require engaging traditional healers, community leaders, and drug regulators alongside pharmacy-level interventions, consistent with calls for broader community-level AMR stewardship in LMICs [37].

Our study had several strengths. Combining in-depth and key informant interviews with an ongoing quantitative study of POC/near-POC testing allowed nuanced exploration of real-time experiences and challenges in using POC/near-POCTs. By involving pharmacy clients, healthcare providers, and policymakers, this study helped capture perspectives across multiple levels of HIV/STI management, thereby enriching the depth and diversity of insights. Interviews were conducted in confidential settings by staff experienced in qualitative research and fluent in both English and Luganda, minimizing social desirability bias and ensuring cultural and linguistic appropriateness. However, the study had some limitations. Treatment and testing were offered for free, contrary to the actual practice. This shows that a co-funded, no-cost approach appears acceptable, although willingness to pay remains unexplored. The high acceptability observed may also reflect ancillary services (counselling, partner notification) not typically available in routine practice. The study was conducted only in private community pharmacies, as Uganda has no standalone public-sector pharmacies; findings are shaped by private-sector dynamics, including the profit motive participants themselves flagged, and may not generalize to a public-sector model, which would face several barriers (wait times, stigma, drug stock-outs). Data were collected only from urban and peri-urban pharmacies, limiting rural applicability. Sensitive HIV/STI subject matter may have introduced social desirability bias, and limited resources precluded back-translation of transcripts, although all were reviewed for accuracy by trained research assistants.

These findings point to recommendations spanning community, provider, and policy levels. At the community level, sensitization campaigns using local media, flyers, and outreach, along with user-friendly, multilingual instructions with visual aids are needed to address literacy-related comprehension gaps. Pharmacists require training in counselling, stigma-reduction communication, test procedures, results interpretation, and linkage to care, alongside regular capacity-building on treatment guidelines and antimicrobial stewardship.

Since current regulations restrict diagnostics to authorized laboratory personnel, future task-shifting models should clearly define appropriate roles for different pharmacy staff cadres. With many private pharmacies in Uganda staffed by registered enrolled nurse dispensers rather than fully qualified pharmacists, this existing workforce could be a practical entry point for task-shifting, as it includes structured training, certification, and supervision, as piloted in South Africa [36]. Given that first-line gonorrhea treatment currently requires intramuscular ceftriaxone, inappropriate oral substitution risks under-treatment and could drive resistance. Competency-based training and certification in injectable antibiotic administration is recommended for any community pharmacy cadre treating gonorrhea and other STIs. Stewardship efforts should engage traditional healers, community leaders, and regulators, given the mixing of antibiotics into unregulated herbal remedies. At a policy level, we recommend integrating community pharmacies into national SRH policies with clear ethical guidelines aligned to HIV/STI protocols, consistent with prior research [12,37], backed by investment in sanitary infrastructure and private consultation areas [36,38]. Further research is needed on the cost-effectiveness of HIV/STI management in community pharmacies to determine whether private pharmacy models could be adapted for public-sector delivery. Priorities for future research also include developing less invasive, more user-friendly POC/near-POC tests supported by digital tools for result interpretation [39], as well as digital surveillance technologies, such as electronic medical records, mobile health applications, AI-powered analytics, and cloud-based data systems, to strengthen antibiotic monitoring and real-time data collection.

### Conclusions

POC/near-POC HIV/STI testing in community pharmacies was perceived as highly acceptable and satisfactory by clients, providers, and policymakers, despite significant challenges around awareness, privacy, infrastructure, and workforce capacity. Realizing this model’s potential will require clearly defined, competency-based task shifting suited to Uganda’s existing pharmacy workforce, treatment protocols that guard against AMR, and multisectoral engagement extending to traditional healers and regulators. Given their accessibility and trusted role in primary care, community pharmacies offer a promising platform for expanding HIV/STI diagnostic access outside traditional clinical settings. Integrated into national health frameworks and informed by further research on public-sector adaptation and cost-effectiveness, this approach could improve early detection and treatment while strengthening surveillance and public health outcomes.

## Supporting information

S1 File Quantitative ‘parent study’ published manuscript. (PDF)

S2 File In-depth Interview guide. (PDF)

S3 File Key Informant Interview guide. (PDF)

S4 File Codebook. (PDF)

## Abbreviations

POC/near-POCTs: Point of care/near-point of care Tests
IDI: In-depth Interviews
KII: Key informant interviews
CPs: Community pharmacies
UNCST: Uganda National Council of Science and Technology
REC: Research Ethics Committee
JCRC: Joint Clinical Research Center
IRB: Institutional Review Board
AMR: Antimicrobial Resistance
STI: Sexually Transmitted Infections
HIV: Human immunodeficiency Virus
MOH: Ministry of Health
