# Supplementary material for: Client, health care provider, and policymaker perspectives on point-of-care/near-point-of-care testing for sexually transmitted infections in community pharmacies in Uganda
Source: PLoS One. 2026 Oct 15;21(10):e0347364. doi: 10.1371/journal.pone.0347364 (PMC13671497; doi:10.1371/journal.pone.0347364)
Supplement: Supplementary file 1 [file pone.0347364.s001.pdf]

PLOS ONE

RESEARCH ARTICLE

Community pharmacies: Key players in point-of-care diagnostics for STI screening in Africa

Agnes N. Kiragga[1,2]*, Annet Onzia[1], Vivian Nakate[1], Irene Bagaya[1], Evelyn Natuha[1], Emmanuel Mande[1], Olivia Kataike[1], Rosalind Parkes-Ratanshi[1,3], Matthew M. Hamill[4☯], Yukari C. Manabe[4☯]

1 Research Department, Infectious Diseases Institute, Makerere College of Health Sciences, Kampala, Uganda, **2** African Population and Health Research Center, Nairobi, Kenya, **3** Academy for Health Innovation, Kampala, Uganda, **4** Division of Infectious Diseases, Department of Medicine, Johns Hopkins University School of Medicine, Baltimore, Maryland, United States of America

☯ These authors contributed equally to this work.
* akiragga@aphrc.org

Check for updates

OPEN ACCESS

Citation: Kiragga AN, Onzia A, Nakate V, Bagaya I, Natuha E, Mande E, et al. (2024) Community pharmacies: Key players in point-of-care diagnostics for STI screening in Africa. PLoS ONE 19(12): e0315191. https://doi.org/10.1371/journal.pone.0315191

Editor: Andrew Max Abaasa, London School of Hygiene and Tropical Medicine Faculty of Epidemiology and Population Health, UGANDA

Received: August 2, 2024

Accepted: November 22, 2024

Published: December 30, 2024

Copyright: © 2024 Kiragga et al. This is an open access article distributed under the terms of the Creative Commons Attribution License, which permits unrestricted use, distribution, and reproduction in any medium, provided the original author and source are credited.

Data Availability Statement: The data have been uploaded as Supplementary information.

Funding: This work was carried out with a grant from the National Institute of Biomedical Imaging and Bioengineering, Grant number 3U54EB007958-15S1, awarded to Dr. Yukari Manabe at Johns Hopkins University, USA. The study funders had no role in the study design, data collection and analysis, the decision to publish, or the preparation of the manuscript.

Abstract

Background

Sexually Transmitted Infections (STIs) rank in the top 5 disease categories for which adults in developing countries seek healthcare services. Community pharmacies offer clients convenience, proximity, extended opening hours, privacy, and efficiency, which could make them desirable locations for HIV and STI screening and treatment. We examined the feasibility of using point-of-care (POC) STI tests for screening HIV and other STIs at community pharmacies.

Methods

We conducted a prospective cohort study of persons seeking medication and other services at 18 purposively selected community pharmacies in Kampala, Uganda. Study participants comprised two broad categories: i) Symptomatic persons aged 18 years who presented with at least one STI sign or symptom and were purchasing treatment for themselves; ii) persons presenting with no STI symptom who had come to purchase any other medication, including family planning services such as emergency contraception. POC tests were used to test HIV, *Chlamydia trachomatis (Ct)*, *Neisseria gonorrhoeae (Ng)*, *Trichomonas vaginalis (Tv)*, and Syphilis. Test results were returned on-site or via telephone within 48 to 72 hours. Descriptive statistics were used to estimate the prevalence of STIs.

Results

Of the 450 participants enrolled, 235 (52.2%) were symptomatic, 215 (47.8%) were asymptomatic, and 280 (62.2%) were females. STI testing was feasible, with an acceptability rate of 99.8%. 135 (30%) of participants had at least one STI; HIV prevalence was *39* (8.7%), Syphilis prevalence was 14 (3.1%), 50 (11.1%) tested positive for Ng, 39 (8.7%) were positive for Ct while. The prevalence of Tv was 25 (8.9%) (tested among women). A total of 107 (23.8%) participants had used an antibiotic in the preceding month.

Competing interests: The authors have declared that no competing interest exist.

Conclusion

Our research underscores the potentially pivotal role of community pharmacies in deploying POC diagnostics for STIs and antimicrobial stewardship by decreasing unnecessary antibiotic dispensation across Africa.

Introduction

The World Health Organization (WHO) estimates an annual global burden of 376 million new infections of four curable sexually Transmitted Infections (STIs): *Chlamydia trachomatis (Ct)*, *Neisseria gonorrhoeae (Ng)*, *Treponema pallidum (TP)*, *Trichomonas vaginalis (Tv)* [1–3], with the highest prevalence in Africa [2, 4]. STIs rank in the top 5 disease categories for which adults in developing countries seek healthcare services (nearly as common as malaria), and the prevalence of STIs is highest among women [1, 5, 6].

In Uganda, the prevalence of curable STIs among women aged 15–24 is estimated at 12.3% for TV, 2.9% for *Ct*, 3.2% for *Tp*, and 5.0% for *Ng* [7]. Among women, the majority of STIs are asymptomatic. STIs have essential sexual, reproductive, and maternal-child health consequences, including genital symptoms, pregnancy complications, infertility, and indirect psychosocial effects [4, 8]. STI control primarily relies on syndromic management, which has very poor specificity and increases the risk of antimicrobial resistance. Bacterial STIs cause genital ulcers and mucosal inflammation that can increase the risk of HIV transmission; increased STI screening and definitive diagnosis will be needed to reach HIV elimination goals. In low- and middle-income countries (LMICs), the prevalence of co-infection of STIs warrants the need for joint screening. In Ethiopia, a recent study reported the prevalence of co-infection of curable STI and HIV remains high in Africa. In Ethiopia, the prevalence of HIV and Syphilis co-infection is 2.2%, while HBV and HIV co-infection is estimated at 5.5% [9]. This warrants the need for combined screening of HIV and other curable STIs.

In low and middle-income African settings, community pharmacies provide primary health care services, including family planning services, supplements, and treatment for fevers and coughs, and these are venues that can be leveraged for HIV and STI screening [10, 11]. A recent systematic review showed that community pharmacies are well-suited to deliver many point-of-care (POC) tests [12]. Community pharmacies offer clients convenience (e.g., short waiting times), proximity to their home or work, extended opening hours, privacy, and efficiency, which could make them desirable locations for HIV and STI screening and treatment. On the contrary, community pharmacies can be a source of misuse of antibiotics if dispensed for treatment of STI symptoms without proper diagnoses [13].

POC diagnostics that meet the World Health Organization's ASSURED (Affordable, Sensitive, Specific, User-friendly, Rapid and robust, Equipment-free, and Deliverable to end-users) criteria [14] provide an opportunity for onsite specimen collection and testing for STIs. Community pharmacies in the United Kingdom [15] and the US have conducted STI screening in retail pharmacies. In Europe, a study conducted in Portugal demonstrated the feasibility of urban community pharmacies as complementary sites for HIV and viral hepatitis screening. They showed that groups with a high risk of infection and lower adherence to screening in health facilities prefer pharmacy testing [16]. In Africa, many studies leverage community pharmacies for HIV and STI screening, with most screening either HIV alone [17] or STIs only.

Data to demonstrate the feasibility of HIV and STI screening in community pharmacies are lacking in sub-Saharan Africa, particularly for persons seeking treatment services in the

community [18]. Outside of HIV, POC STI testing in sub-Saharan Africa generally remains low, and their application in settings outside of clinics to reach potentially high-risk populations warrants exploration. Furthermore, exploring the feasibility of specimen self-collection at community pharmacies (i.e., urine, vaginal swabs) [19, 20] is essential to reduce barriers to testing further. Therefore, we aimed to examine the feasibility of using POC STI tests for screening HIV and other curable STIs among symptomatic and asymptomatic persons attending community pharmacies in urban Uganda, including estimating their prevalence and incidence.

Methods

Ethical statement

The study received ethics approval from the Joint Clinical Research Center (JC1820), the Uganda National Council for Science and Technology (HS1274ES), and the Johns Hopkins Institutional Review Board (IRB: 00279241). All adult and emancipated minor study participants provided written informed consent before participating.

Study design and settings

The study was conducted between 05/15/2021 and 11/31/2023, led by researchers from the Infectious Diseases Institute (IDI) at Makerere University in Kampala, Uganda. We conducted a prospective cohort study of persons or clients seeking medication and other services at community pharmacies in Kampala, Uganda. A community pharmacy, sometimes called a retail pharmacy, is a dedicated store or drug outlet where all the prescribed and compounded medications are sold. A pharmacy provides health information, should only dispense drugs upon prescription, and must be done by a licensed pharmacist [21]. A total of 18 pharmacies were purposively selected from all five administrative divisions of the Kampala district. The pharmacies were selected if their own or the proprietor agreed to have the study conducted on his premises, there was ample space for the research team to conduct their activities, there was availability of a nearby bathroom for sample collection, and the weekly clientele was at least 100 per week. Study participants comprised two broad categories: i) Symptomatic persons who included men and women aged 18 years and above or emancipated minors, who presented with at least one STI sign or symptom (genital discharge, genital ulceration, rectal discharge, rectal pain, abdominal pain, testicular/scrotal pain, rash) and were purchasing treatment for themselves. We used consecutive sampling to enroll all persons who agreed to participate and provided consent. ii) persons presenting with no STI symptoms who had come to purchase any other medication, including family planning services such as emergency contraception. We used systematic sampling; every third person who agreed to participate in the study and provided consent was enrolled.

All participants were willing to be followed up at monthly intervals for three months to assess clinical treatment response/failure for persons diagnosed with an STI and incident infection from study enrolment. During follow-up, participants who were diagnosed with HIV were followed up to access linkage to care for HIV and Pre-exposure prophylaxis (PrEP services if eligible. To minimize the risk of breaching participants' confidentiality and to prevent the unnecessary disclosure of individuals' HIV or STI status, no personal identifiers were included on the study Case Report Forms (CRF) or any related documents. All specimens collected during HIV and STI tests were anonymous and were assigned unique study codes, ensuring that they could not be linked to specific participants.

Sample collection and STI testing. All participants provided consent for sample collection and storage for future tests. The Ethics Committee approved the consent. Specimen

collection was conducted in a safe, secure environment within the community pharmacy. This was in. a mobile tent set up near the pharmacy and accessible only to the study team and study participants. A trained research study nurse collected venous blood (5 ml of plasma and 5 ml of serum) from each participant using an aseptic technique. Whole EDTA blood (10 ul) was applied to the HIV-syphilis POC tests (SD Bioline, Abbott, city, country) at the community pharmacy.

In contrast, the remaining blood was transported to the IDI translational lab for additional reference laboratory testing and future storage. Participants were asked to self-collect three vaginal swabs for women and three penile meatal swabs for men for Ng/Ct, trichomoniasis (only tested for females), and one additional swab for storage. The Gene Xpert (Cepheid, HBDC, Maurens-Scopont, France) was used for testing Ng and Ct testing, and the Osom Sekisui Diagnostics LLC, San Diego, CA) for POC trichomonas test was used (for men with urethritis only).

Results notification. HIV, syphilis, and Tv results were returned to all the participants who preferred to wait for results at the pharmacy. For subsequent dispatch of results or any other study-related issue over the phone, the participants were asked to confirm a unique access code assigned to each participant at enrollment. This measure helped prevent unintentional disclosure of participants' information, particularly when sharing mobile phones. The Ng/Ct results were returned within 24 hours, and the Ng culture and sensitivity results were returned within 48 to 72 hours via the telephone number provided at the study enrolment. All participants who tested positive for any STI were asked to share contacts of their partners and consent to notify them of their status and encourage them to come for STI testing and treatment. A nested study compared the effect of using the Call for Life Telehealth Platform for partner notification with the standard of care [22]. The results will be published in a separate manuscript.

STI and HIV treatment

All study participants who tested positive for HIV were counseled and immediately referred to the nearest national HIV care treatment public facility for HIV confirmation and initiation of antiretroviral therapy. Treatment was based on the laboratory-identified STI following the Ugandan STI guidelines. Participants who tested positive onsite for any curable STI at the pharmacy and those who received their test results over phone calls were asked to return to the pharmacy to receive treatment or a prescription to enable them to access treatment at an alternative pharmacy or national STI treatment center. Treatment for all STIs followed the guidelines from the Ministry of Health Uganda and the Centers for Disease Control and Prevention [23].

Data collection methods

Participants were recruited and completed data collection between May 2021 and June 2022. A structured questionnaire designed in REDCap (Vanderbilt, Tennessee, USA), was administered to eligible participants by research nurse counselors at the pharmacy. Information collected included demographic and social characteristics, medical and medication history, sexual behavior patterns, STI knowledge and testing history, and alcohol use. Participants were also assessed for their willingness to use HIV Pre pre-exposure prophylaxis (PrEP) services if they were eligible per country guidelines.

Statistical methods

Descriptive statistics, including means and medians, were used to describe the study population in the two groups. Simple statistics tests, including Chi-square tests, were used to test for

differences in proportions of participant characteristics with their willingness to use PrEP, T-tests, and Mann-Whitney rank sum tests for normally and non-normally distributed continuous variables. Associations between baseline characteristics, sexual behavior, presence of STI symptoms, and curable STI diagnoses were described. All analysis was performed in STATA 17.0.

Results

Feasibility of STI diagnosis using POC devices at community pharmacies

Between May 2021 and June 2022, a total of 474 persons who were seeking treatment for STI-like signs and symptoms (*symptomatic group*) and other family planning services such as emergency contraception for self or partner (*asymptomatic group*) were approached for participation. Of these, 24 were excluded at screening (Fig 1). A total of 450 persons were enrolled from 18 community (retail) pharmacies in Kampala City, the largest peri-urban district in Uganda. Of the 450 participants, 235 (52.2%) were symptomatic, 215 (47.8%) were asymptomatic, and 62.2% (n = 280) were females. More males were symptomatic, 103 (43.8%), than asymptomatic, 67 (31.2%), P = 0.006. Educational attainment was lower among the symptomatic group, with 78 (33.2%) having attained at most primary level education, compared to 42 (19.5%) in the asymptomatic group, P = 0.001, Table 1. The majority of participants were heterosexual. However, 15.7% of symptomatic and 10.2% of asymptomatic participants reported

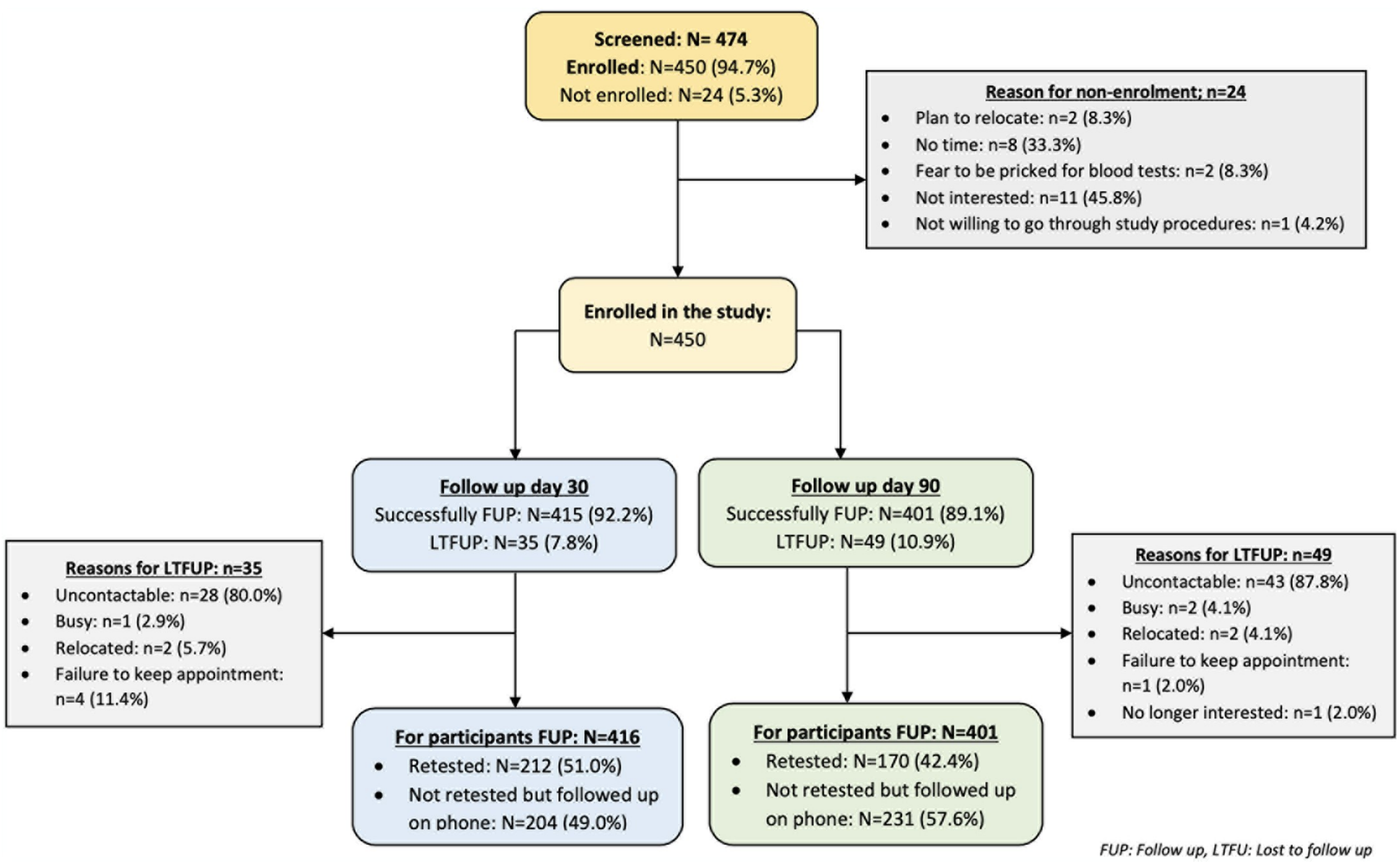

Fig 1. Figure shows the study population, including STI asymptomatic and symptomatic clients seeking services at community pharmacies in Kampala, Uganda.

https://doi.org/10.1371/journal.pone.0315191.g001

Table 1. Demographic and clinical characteristics of STI asymptomatic and symptomatic clients seeking services at community pharmacies in Kampala, Uganda.

	**Total**	**Study group, frequency (%)**		**P-Value**
	**N = 450 (100.0) *n (col%)***	**Asymptomatic**	**Symptomatic**	
		**N = 215 (47.8) n (col%)**	**N = 235 (52.2) *n (col%)***	
**Age in years (Median (IQR)**	28 (24–35)	27 (24–34)	28 (24–37)	0.365[c]
≤24 years	126 (28.0)	60 (27.9)	66 (28.1)	
25–34 years	203 (45.1)	102 (47.4)	101 (43.0)	
≥35 years	121 (26.9)	53 (24.7)	68 (28.9)	
**Gender:** Male	170 (37.8)	67 (31.2)	103 (43.8)	**0.006**
Female	280 (62.2)	148 (68.8)	132 (56.2)	
**Marital status:** Married	222 (49.3)	109 (50.7)	113 (48.1)	0.097
Single with no regular partner	37 (8.2)	11 (5.1)	26 (11.1)	
Single with regular partner	175 (38.9)	89 (41.4)	86 (36.6)	
Separated/widow	16 (3.6)	6 (2.8)	10 (4.3)	
**Education:** Above Primary	330 (73.3)	173 (80.5)	157 (66.8)	**0.001**
Primary & below	120 (26.7)	42 (19.5)	78 (33.2)	
**Employment type:** Formal	201 (44.7)	102 (47.4)	99 (42.1)	0.472
Self	177 (39.3)	82 (38.1)	95 (40.4)	
None	72 (16.0)	31 (14.4)	41 (17.5)	
**Number of sex partners:** None	7 (1.6)	3 (1.4)	4 (1.7)	**<0.001**
1 partner	336 (74.7)	179 (83.3)	157 (66.8)	
2+	107 (23.8)	33 (15.3)	74 (31.5)	
**Condom use:** Always	20 (4.4)	10 (4.6)	10 (4.3)	0.924
Sometimes	135 (30.0)	66 (30.7)	69 (29.4)	
Never	295 (65.6)	139 (64.7)	156 (66.4)	
**Sex orientation:** Heterosexual	391 (86.9)	193 (89.8)	198 (84.3)	0.136[f]
Homosexual	55 (12.2)	20 (9.3)	35 (14.9)	
Bisexual	1 (0.2)	0	1 (0.4)	
Unknown	3 (0.7)	2 (0.9)	1 (0.4)	
**Engage in transactional sex**	239 (53.1)	120 (55.8)	119 (50.6)	0.695
**STI Knowledge: High**	229 (50.9)	116 (54.0)	113 (48.1)	0.214
Low or none	221 (49.1)	99 (46.0)	122 (51.9)	
**Willingness to take prophylactic STI treatment**	428 (95.1)	203 (94.4)	225 (95.7)	0.515
**Willing to inform partner of STI results**	400 (88.9)	199 (92.6)	201 (85.5)	**0.018**
**Illicit drug use in past 6 months**	16 (3.6)	5 (2.3)	11 (4.7)	0.178
**Alcohol in the last 12 months**	119 (26.4)	45 (20.9)	74 (31.5)	**0.011**
**Antibiotics use in past month**	107 (23.8)	30 (13.9)	77 (32.8)	**<0.001**[f]
**Diagnosed with an STI**	315 (70.0)	176 (81.9)	139 (59.1)	**<0.001**

Column percentages are presented. P-values were obtained using Pearson chi-square test except [c]P-value by rank-sum test used to compare median age, P-value* by fishers exact

• STI symptom present[1] considered participant with any symptoms including (Urethral pus discharge, Abnormal vaginal discharge, Genital swelling or Genital growth, Lower abdominal pain, Genital itching)

• Have STI considered participants with any sexually transmitted infection i.e. HIV, Syphilis (using Abbott Bio-line Duo), Gonorrhea, Chlamydia (using Cepheid GeneXpert) Trichomoniasis (using OSOM lateral flow assay) from confirmatory laboratory tests.

https://doi.org/10.1371/journal.pone.0315191.t001

same-sex or bisexual activities. S1 Table shows the demographic and social characteristics breakdown by STI status at study enrollment.

Feasibility of self-sample collection

Sample collection by laboratory technicians for blood and self-sample collection of genital swabs was feasible and acceptable. Out of 450 participants, only one declined blood sample provision for on-site HIV and syphilis testing, resulting in a 99.8% acceptability rate. Additionally, 100% of participants provided self-collected swabs (penile meatal in men, vaginal in women) for Ct/Ng testing as well as Trichomonas lateral flow testing. During follow-up visits on days 30 and 90, 47.1% and 37.8% of participants, respectively, underwent repeat tests. Two participants declined testing for Ng and Ct on day 30 due to the inability to go through the process. All study participants chose to wait for the results, and the average turnaround time for on-site POC tests (HIV, Syphilis, Trichomonas) was an average of 15 minutes. Specimens transported for the GeneXpert test took an average of 100 minutes, including transportation via a motorbike. Overall, the feasibility of accurate POC testing was demonstrated by the proportion of clients who waited for their HIV, syphilis, and Trichomonas results, which took approximately 20 minutes.

Behaviors associated with STIs and STI knowledge. Twice as many participants in the symptomatic group had multiple (two or more) partners in the previous 6 months, 74 (31.5%), compared to the asymptomatic group 33 (15.3%), $P<0.001$.

Condom use was generally low; 295 (65.6%) reported never using a condom. There were no significant differences in condom use between the symptomatic and asymptomatic groups. A total of 239 (53.1%) participants had engaged in some form of transactional sex in the past 6 months. Overall, 119 (26.4%) have consumed alcohol in the past 12 months, and alcohol consumption was higher among the symptomatic participants at 74 (31.5%) compared to 45 (20.9%) among asymptomatic participants, $P = 0.011$.

Only half, 229 (50.9%), had good knowledge (knew at least two correct ways of STI transmission and prevention) of STIs and how they are transmitted and treated. When asked whether the participants were willing to take STI prophylactic treatment (e.g., doxycycline) if considered high-risk, 428 (95.1%) were willing. Most participants, 400 (88.9%), were willing to disclose the results from the STI test to their partners. However, a higher proportion of symptomatic participants, 34 (14.5%) compared to 16 (7.4%), $P = 0.018$, were unwilling to disclose the results of their STI testing to their respective sexual partners. The use of antibiotics was reported among the study participants. Nearly one in four participants, 107 (23.8%), has used an antibiotic in the preceding 30 days. The proportion was higher among the symptomatic group, 77 (32.8%), compared to the 30 (13.9%) asymptomatic, $P<0.001$.

Prevalence of STIs

In the study population, 135 (30%) of participants had at least one STI; 96 (40.9%) were symptomatic versus 39 (18.1%) in asymptomatic participants, $P<0.001$, Table 2. The prevalence of STIs was higher among men 67/170 (39.4%) compared to women 68/280 (24.3%), $P<0.001$, Fig 2. The proportion of symptomatic participants with at least one STI was 40.8% compared to 18.6% among asymptomatic, $P<0.001$. Among the symptomatic participants, 74 (31.5%) had one STI, and 22 (9.3%) had two or more STIs. In the asymptomatic group, 35 (16.3%) had only one STI, while 5 (2.2%) had at least two STIs.

HIV. *Prevalence*: Among the 450, 39 (8.7%) tested HIV-positive, and of these, 27 (69.2%) were aware of their status (22 of 27 (81.5%) were taking antiretroviral therapy); 12 (30.8%) were newly diagnosed. HIV prevalence was not different between males 39/170 (8.7%) and

Table 2. Sex disaggregated prevalence of Sexually Transmitted Infections (STIs) among STI asymptomatic and symptomatic clients seeking services at community pharmacies in Kampala, Uganda.

STI	Total, N = 450 N (%)	Male, n = 170 N (%)	Female, n = 280 N (%)	P Value
Any STI	135 (30.0)	67 (39.4)	68 (24.3)	**<0.001**
HIV	39 (8.7)	15 (8.8)	24 (8.6)	0.927
Syphilis	14 (3.1)	4 (2.3)	10 (3.6)	0.470
Neisseria gonorrhoeae (Ng)	50 (11.1)	40 (23.5)	10 (3.6)	**<0.001**
Chlamydia trachomatis (Ct)	39 (8.7)	19 (11.2)	20 (7.2)	0.140
Trichomonas*	25 (8.9)	N/A	25 (8.9)	

*Only tested among females

https://doi.org/10.1371/journal.pone.0315191.t002

females 15 (8.8%), P = 0.927, Table 2. The prevalence of HIV was two-fold higher, 11.5% among the symptomatic group, compared to the asymptomatic, 5.6%, P = 0.027, Fig 3. Out of 410 who were HIV-negative or not aware of their HIV status, 266 (62.7%) were female; 298 (70.3%) were 25 years and above; 209 (49.3%) were married; the majority, 312 (73.6%) had above primary level education; 322 (75.9%) reported to have a sex partner in the previous six months; 279 (65.8%) reported to never using condoms; 222 (52.4%) had a history of engagement in transactional sex; 297 (70.0%) had never heard of PrEP.

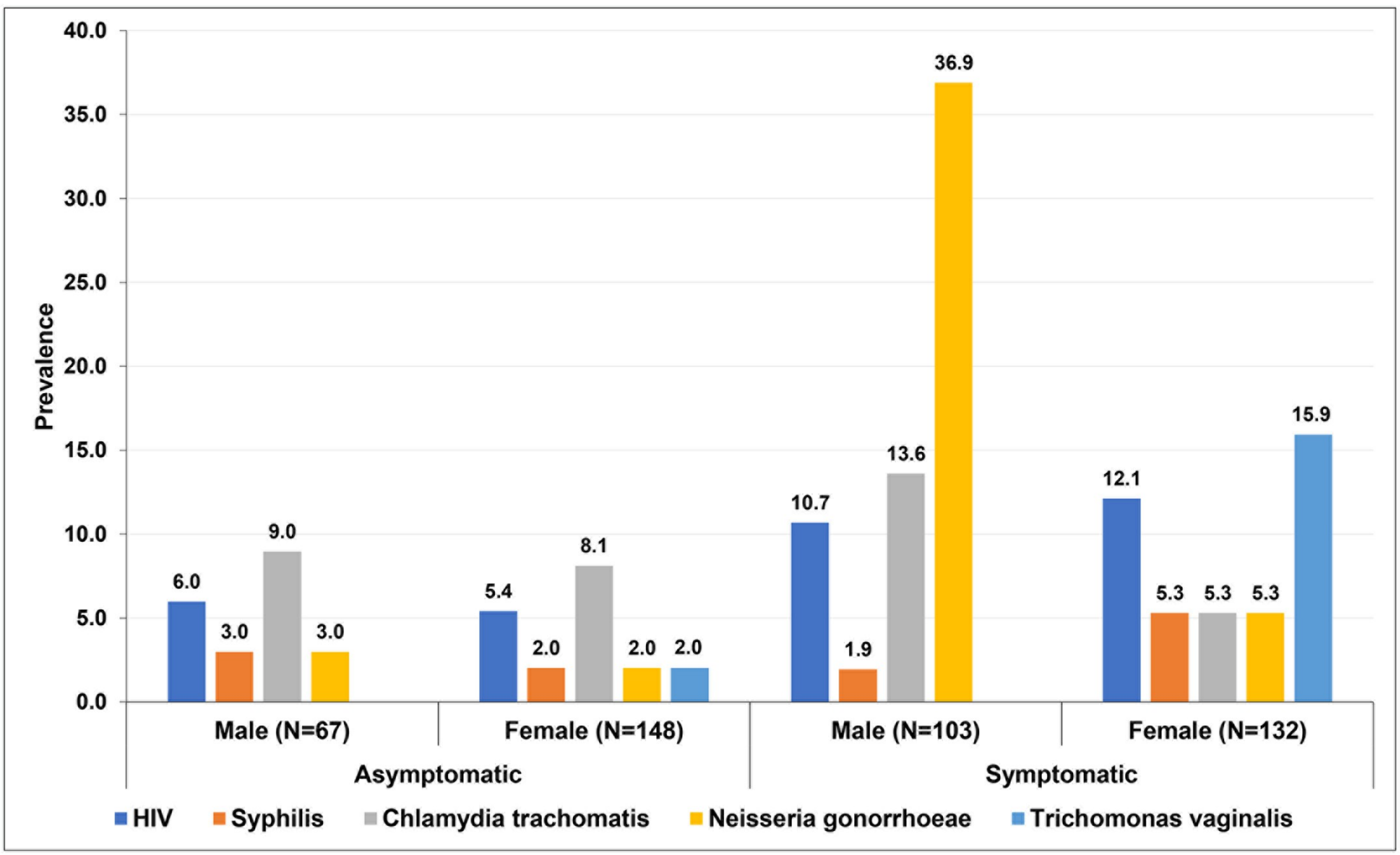

Fig 2. Overall prevalence of HIV, Syphilis, Chlamydia trachomatis, Neisseria gonorrhoeae, and Trichomonas vaginalis among clients seeking services at community pharmacies in Kampala, Uganda.

https://doi.org/10.1371/journal.pone.0315191.g002

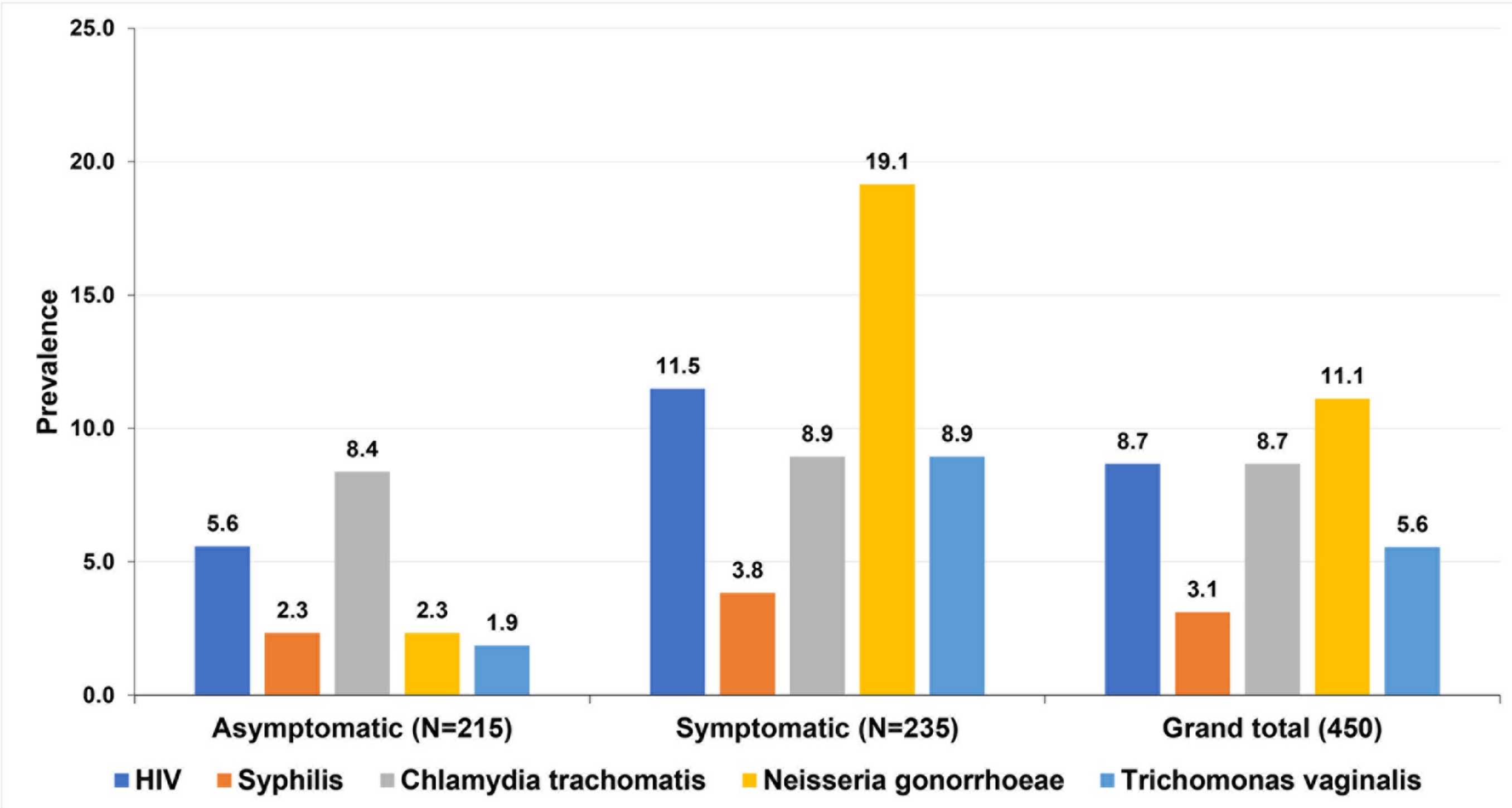

Fig 3. Sex-disaggregated prevalence of HIV, Syphilis, Chlamydia trachomatis, Neisseria gonorrhoeae, and Trichomonas vaginalis among asymptomatic and symptomatic clients seeking services at community pharmacies in Kampala, Uganda.

https://doi.org/10.1371/journal.pone.0315191.g003

Incidence. At the day 30 visit, among the 212 participants who were retested, one (0.5%) male participant (who initially came to the pharmacy to purchase STI treatment) was newly diagnosed with HIV. On day 90, one additional incident HIV case was identified. The two participants who were seroconverted were males (aged 18–30 years), one with multiple sexual partners and the other engaged in transactional sex. One tested positive for syphilis at study enrolment, Table 3.

Table 3. Prevalent and incident STIs among STI asymptomatic and symptomatic clients seeking services at community pharmacies in Kampala, Uganda.

	Baseline, n = 450		Day 30, n = 212			Day 90, n = 170		
**STI results, n (%)**	**Positive**	**Negative**	**Positive**	**Negative**	**Incident cases**	**Positive**	**Negative**	**Incident cases**
HIV	39 (8.7)	410 (91.3)	24 (11.3)	187 (88.2)	1 (0.5)	15 (8.8)	154 (90.6)	1 (0.6)
Syphilis	14 (3.1)	434 (96.7)	9 (4.2)	199 (93.9)	4 (1.9)	5 (2.9)	165 (97.1)	0 (0.0)
Neisseria gonorrhoeae* (GeneXpert) (NG)	50 (11.1)	400 (88.9)	5 (2.4)	203 (97.1)	1 (0.5)	2 (1.2)	165 (97.0)	3 (1.8)
Chlamydia trachomatis (CT)	39 (8.7)	410 (91.1)	3 (1.4)	204 (97.2)	3 (1.4)	3 (1.8)	166 (97.6)	1 (0.6)
Trichomonas*	25 (8.9)	255 (91.1)	2 (1.6)	126 (98.4)	0 (0.0)	0 (0.0)	96 (100.0)	0 (0.0)

Note:

*Trichomonas was tested only among women (n = 280), day 30 (n = 128), day 90 (n = 96)

Baseline: Trichomonas was tested only among women (n = 280). Two participants had indeterminate results for Syphilis and Chlamydia trachomatis; 1 participant refused to be bled for HIV& syphilis at baseline. Day 30: day 30 (n = 128), day 90 (96): NG & CT: day 30 (n = 210); 2 participants declined to retest for Ng & Ct at day 30. Day 90: One participant had an indeterminate result for NG on day 30.

https://doi.org/10.1371/journal.pone.0315191.t003

Syphilis. *Prevalence*. Among 438 participants with evaluable results, 14 (3.1%) had a positive treponemal antibody POCT; 7 (50%) participants had reactive rapid plasma reagin (RPR) titers, (1:1 [n = 1], 1:2 [n = 2], 1:8 [n = 2], 1:16 [n = 1], 1:32 [n = 1] and 1:1 [n = 1]. All were staged as late latent syphilis. Most cases (9/14) were in the symptomatic group, and 7/9 were females. At day 30, 3/6 (50%) had cleared their syphilis; one had unresolved syphilis with titers at 1:4, while two had missing titers. At day 90, the client with unresolved syphilis at day 30 had active syphilis with titers estimated at 1:4. *Incidence*. At day 30, four new syphilis cases were identified by POCT, a prevalence rate of 4.2%, and all were staged as early latent syphilis. Two of the incident cases were females who came to the pharmacy to purchase emergency contraception and STI treatment, Table 3. Out of 13 Participants who tested positive for Syphilis at day 30, 8 were retested at day 90, and 3/8 (37.5%) participants had their infection resolved. On day 90, there were no incident cases of syphilis.

Neisseria gonorrhoeae (Ng)

Prevalence: 50 (11.1%) of the study population tested positive for Ng. The majority, 40 (80%), were males, and 45/50 (90%) were symptomatic and seeking STI treatment at the pharmacy. *Incidence*: On day 30, 210 participants were retested for Ng; one symptomatic male participant (0.5%) was diagnosed with a new infection, 30/50 positive Ng results were retested, and 5/30 (16.7%) had unresolved infections diagnosed at baseline. At the 90-day visit, 170 participants were retested; 3 (1.8%) new cases were diagnosed, while 2 (1.2%) were unresolved diagnosed cases. *Resolution*. Out of 50 Participants who tested positive for Ng at baseline, 30 were retested on day 30, and 25/30 (83.3%) participants had resolved their infection. On day 90, out of 6 participants who tested positive for Ng on day 30, 4 had results on day 90, and 3/4 (75%) had a resolved Ng infection, Table 3.

Chlamydia trachomatis (Ct)

Prevalence: At baseline, 39 (8.7%) participants tested positive for Ct, 20 (51.3%) were females, and 21/39 (53.9%) were asymptomatic. *Incidence*: On day 30, 3 (1.4%) new cases of Ct were identified (two females and one male participant seeking STI treatment). On day 90, one female participant seeking STI treatment tested positive 1 (0.6%). *Resolution*: Out of 39 participants who tested positive for CT at baseline, 25 had repeat tests on day 30, and 22/25 (88.0%) had resolved. Out of 6 participants who tested positive for CT on day 30, 4 had repeat tests on day 90, and 3/4 (75%) had their infection resolved Table 3.

Trichomonas

Prevalence: Of 280 women tested for Trichomonas, 25 (8.9%) tested positive. The majority, 21 (84%), were seeking treatment. *Incidence*: On day 30, 3 (1.4%) incidents were observed. At day 90, no female participant tested positive. *Resolution*: Out of 25 participants who tested positive for Trichomonas at baseline and attended for follow-up, 14 were retested on day 30, and 12/14 (85.7%) had resolved infection, Table 3.

Discussion

Our research underscores the potentially pivotal role of community pharmacies in deploying POC diagnostics for sexually transmitted infections (STIs) across Africa. Given the challenges in accessing traditional healthcare facilities due to distance and resource constraints, community pharmacies emerge as vital hubs for timely diagnostic services and treatment, particularly for less severe ailments. Our findings demonstrate the feasibility of STI testing in these settings,

with an overwhelming majority (99%) of participants consenting to and providing adequate specimens onsite. This underscores the importance of on-site POC testing in delivering prompt STI results to symptomatic and asymptomatic individuals.

Moreover, the availability of POC STI tests presents a promising avenue for combatting the misuse of antibiotics. Our study reveals a concerning trend of antibiotic use without a prescription, highlighting the urgent need for interventions. With community-based diagnostics offering swift results, antibiotic misuse could be replaced with appropriate prescriptions based on accurate etiological diagnoses. This approach may offer another advantage of being cheaper if specific drugs are used, compared to syndromic treatment, where more expensive broad-spectrum drugs target all possible organisms. This holds significant promise in mitigating antimicrobial resistance, thus safeguarding public health in Africa.

Community pharmacies play a crucial role in reaching both asymptomatic and symptomatic individuals at high risk of HIV and STI transmission within the community. Our study revealed that approximately one in three individuals had at least one STI, with the majority exhibiting symptoms but not seeking care at hospitals. Instead, they sought medication for undiagnosed infections from pharmacists or drug dispensers based on symptoms. These individuals constitute a population at high risk who would be eligible for PrEP services if HIV they are uninfected. Community pharmacies, being accessible and trusted healthcare settings, are well-positioned to provide essential services such as screening, education, and dispensing of medications, contributing significantly to HIV and STI prevention efforts.

A recent systematic review underscored the potential of community pharmacies for POC testing, covering various ailments such as blood glucose, cholesterol, liver enzymes, and HIV [12]. While the reviewed studies did not encompass screenings for other curable sexually transmitted infections (STIs), including those examined in our research, they highlighted the suitability of community pharmacies in delivering diverse, high-quality services [12]. Additionally, evidence from New York suggests that community pharmacies play a vital role in HIV testing among high-risk populations, complementing national strategies for HIV prevention [24]. This indicates the potential of leveraging community pharmacies not only for HIV testing but also for expanding STI screening services to enhance public health outcomes. This is further justified by the high prevalence of persons living with HIV and co-infected with other curable STIs, such as syphilis, as reported in Africa and the broader global community [9, 25]. However, this should be done cautiously to avoid misuse of antibiotics when dispensed without prescriptions. This warrants the continuous rigorous training of drug dispensers to support antimicrobial stewardship.

Our study sheds light on missed opportunities for HIV prevention, particularly in programs not implemented at the community level. Within the study, out of 39 persons who tested positive for HIV, a third (n = 12) did not know their status. This has implications for increased awareness of HIV testing, if we are to achieve the United Nations 95-95-95 goals, specifically ensuring that 95% of persons are aware of their HIV status. Furthermore, two individuals at risk for HIV, characterized by factors such as multiple sexual partners, engagement in transactional sex, and testing positive for syphilis, HIV seroconverted 30 and 90 days after their initial HIV screening at the community pharmacy. This underscores the syndics of bacterial STIs and HIV. The findings highlight the significance of early intervention strategies at the community level to mitigate HIV transmission risks. By identifying and addressing missed opportunities for prevention, community-level interventions can play a crucial role in reducing the incidence of HIV transmission.

The prevalence of HIV and other STIs in our study aligns closely with estimates from the Uganda Population-Based HIV Impact Assessment (UPHIA) 2029–2021 [26]. In the UPHIA survey, Kampala and Wakiso districts reported HIV prevalence of 6.0% and 8.1%, respectively.

Similarly, our study observed an HIV prevalence of 8.7%, indicating consistency with UPHIA estimates [26]. Additionally, our study's syphilis prevalence of 3.1% mirrors the UPHIA estimates and findings from a similar survey conducted in gaming centers in Kampala [27]. Furthermore, our study identified incident syphilis at day 30 and day 90 post-enrolment, suggesting the potential utility of doxycycline for STI prevention in individuals at risk, particularly young men [28]. The prevalence of the other curable STIs, including Neisseria Gonorrhea (Ng), Chlamydia trachomatis (Ct), and Trichomonas, was comparable with global estimates for Uganda [29]. We observed differences in education status by STI symptomatic status, with slightly higher education levels found among asymptomatic patients. This suggests that education may play a role in health literacy, enabling better-informed patients to seek care earlier than those with less education. This finding is significant and highlights the growing interest in understanding social determinants of health, such as education, and their impact on well-being and life expectancy.

Our study highlighted the feasibility and significance of POC screening for HIV and other sexually transmitted infections (STIs) at community pharmacies in urban areas. The robust uptake of these services among community pharmacy clients reflects their eagerness to ascertain their health status promptly and conveniently. Community pharmacies offer a non-stigmatized environment and serve as comprehensive healthcare hubs, facilitating access to various services beyond sexual health. Pre-test counseling and early detection of HIV and STIs foster prevention behaviors and empower individuals with heightened sexual health awareness. These findings advocate for expanding community pharmacies' roles in LMICs and addressing healthcare access challenges. Implementing POC screening services in pharmacies can be pivotal in bridging gaps in healthcare delivery, particularly in regions facing resource limitations. However, pharmacists need to be fully onboarded, specifically if they are to forego the potential profits made from the empiric treatment of STIs versus diagnosed infections, half of whom could have been over-treated with antibiotics.

Despite the demonstrated feasibility, the implementation of HIV and other STI screening or testing at the POC in community pharmacies faces significant challenges. Primarily, the availability of suitable environments for sample collection in urban settings poses a considerable hurdle. Obtaining permission from pharmacy proprietors to conduct sample collection activities and ensuring the provision of safe and private spaces for participants adds complexity. Moreover, collaboration with public health officials and obtaining approvals from relevant regulatory bodies, such as the Ministry of Health, is imperative for future studies and scaling up screening efforts. Additionally, identifying alternative venues within communities for STI testing, particularly to reach demographics with higher-risk behaviors, such as young men, is essential for enhancing access to screening services. Addressing these challenges is crucial for the successful implementation and scalability of HIV and STI screening initiatives in community pharmacy settings.

The study site and participant selection processes may have introduced bias. The community pharmacies selected for this study were located in urban and peri-urban districts, which may not represent all pharmacies across the country, particularly those in rural areas. Additionally, purposive sampling was used to ensure that the pharmacies had the necessary infrastructure to support the study, such as a testing area and a nearby bathroom for sample collection. The clientele visiting these pharmacies may have different financial capabilities than those not using the study pharmacies. While information about the study was provided to all pharmacy clients, pharmacists might have selectively referred patients based on the perceived severity of symptoms. This could result in a skewed representation of participants, potentially affecting treatment outcomes and cost considerations. This was especially the case when pharmacists were reluctant to refer clients from whom they could make the most profit

via direct over-treatment with antibiotics and other medication. Notably, a significant loss to follow-up was observed on days 30 and 90 of the study. This was partly driven by the fact that some respondents were transient, unlike to return to the same pharmacy, and unavailable after enrolment. This could have led to differences between participants who continued versus those who dropped out, impacting subsequent retesting and potentially influencing estimates of STI incidence. Differences in engagement with retesting, particularly among individuals engaging in high-risk behaviors, could further contribute to selection bias. These potential sources of selection bias underscore the importance of robust participant recruitment strategies and addressing attrition challenges to ensure the validity and reliability of study findings. Lastly, the costs of POC tests are high and unaffordable in LMICs. A 10-pack of Xpert NG/CT cartridges costs approximately USD160, and 25 OSOM TV tests cost 80USD, which is out-of-reach for retail pharmacists who would prefer to have a diagnosis made before providing antibiotics. This impacts the scale-up of true POC diagnoses for STIs such as Ng and Ct. Furthermore, the turnaround time of 90 minutes is too long for a clinical encounter; participants were willing to wait 15 minutes for the rapid lateral flow tests. Tests for Ng/Ct will need to be shorter to ensure timely treatment.

Conclusion

Community pharmacies, in conjunction with POC diagnostics, offer a feasible and effective alternative for HIV and STI screening, testing, treatment, and prevention. This strategy enables the detection of STIs, which could otherwise contribute to community transmission, especially among women experiencing milder STI symptoms and young men engaging in high-risk behaviors and relying on self-medication from pharmacies. Moreover, integrating STI screening and testing into pharmacy services promises antimicrobial stewardship by decreasing unnecessary antibiotic dispensation. However, to ensure the successful expansion of STI testing in community pharmacies, the Ministry of Health must provide support and regulatory oversight, ensuring adherence to established guidelines and standards. We recommend the development of partnerships and policies that recognize Community Retail Pharmacies for STI prevention in Uganda. Such policy can leverage current partnerships with the community pharmacies, such as the Community Retail Pharmacy Drug Distribution Points model in Uganda [30].

Supporting information

S1 Table. STI prevalence by demographic characteristics, sexual behavior, STI knowledge and symptom status.
(PDF)

Author Contributions

Conceptualization: Agnes N. Kiragga, Yukari C. Manabe.

Data curation: Vivian Nakate, Evelyn Natuha, Emmanuel Mande, Olivia Kataike.

Formal analysis: Agnes N. Kiragga, Vivian Nakate, Rosalind Parkes-Ratanshi.

Funding acquisition: Agnes N. Kiragga, Rosalind Parkes-Ratanshi, Yukari C. Manabe.

Methodology: Yukari C. Manabe.

Project administration: Agnes N. Kiragga, Annet Onzia, Irene Bagaya, Evelyn Natuha, Olivia Kataike.

Supervision: Agnes N. Kiragga, Annet Onzia, Rosalind Parkes-Ratanshi, Matthew M. Hamill, Yukari C. Manabe.

Visualization: Vivian Nakate, Emmanuel Mande, Matthew M. Hamill.

Writing – original draft: Agnes N. Kiragga.

Writing – review & editing: Annet Onzia, Vivian Nakate, Irene Bagaya, Evelyn Natuha, Emmanuel Mande, Olivia Kataike, Rosalind Parkes-Ratanshi, Matthew M. Hamill, Yukari C. Manabe.

References

1. Organization; WH. Sexually transmitted infections (STIs). http://www.who.int/mediacentre/factsheets/fs110/en/ (accessed 28 January 2020).
2. Newman L, Rowley J, Vander Hoorn S, Wijesooriya NS, Unemo M, Low N, et al. Global Estimates of the Prevalence and Incidence of Four Curable Sexually Transmitted Infections in 2012 Based on Systematic Review and Global Reporting. PLoS One. 2015; 10(12):e0143304. https://doi.org/10.1371/journal.pone.0143304 PMID: 26646541
3. Zheng Y, Yu Q, Lin Y, Zhou Y, Lan L, Yang S, et al. Global burden and trends of sexually transmitted infections from 1990 to 2019: an observational trend study. Lancet Infect Dis. 2022; 22(4):541–51. https://doi.org/10.1016/S1473-3099(21)00448-5 PMID: 34942091
4. Organization; WH. Sexually Transmitted Infections: Key Facts. https://wwwwhoint/news-room/fact-sheets/detail/sexually-transmitted-infections-(stis)#:~:text=More%20than%201%20million%20sexually,%2C%20gonorrhoea%2C%20syphilis%20and%20trichomoniasis. 2022.
5. Chico RM, Mayaud P, Ariti C, Mabey D, Ronsmans C, Chandramohan D. Prevalence of malaria and sexually transmitted and reproductive tract infections in pregnancy in sub-Saharan Africa: a systematic review. JAMA. 2012; 307(19):2079–86. https://doi.org/10.1001/jama.2012.3428 PMID: 22665107
6. Gaydos CA, Klausner JD, Pai NP, Kelly H, Coltart C, Peeling RW. Rapid and point-of-care tests for the diagnosis of Trichomonas vaginalis in women and men. Sex Transm Infect. 2017; 93(S4):S31–S5. https://doi.org/10.1136/sextrans-2016-053063 PMID: 28684611
7. Rowley J, Vander Hoorn S, Korenromp E, Low N, Unemo M, Abu-Raddad LJ, et al. Chlamydia, gonorrhoea, trichomoniasis and syphilis: global prevalence and incidence estimates, 2016. Bull World Health Organ. 2019; 97(8):548–62P. https://doi.org/10.2471/BLT.18.228486 PMID: 31384073
8. Tsevat DG, Wiesenfeld HC, Parks C, Peipert JF. Sexually transmitted diseases and infertility. Am J Obstet Gynecol. 2017; 216(1):1–9. https://doi.org/10.1016/j.ajog.2016.08.008 PMID: 28007229
9. Getaneh Y, Getnet F, Amogne MD, Liao L, Yi F, Shao Y. Burden of hepatitis B virus and syphilis co-infections and its impact on HIV treatment outcome in Ethiopia: nationwide community-based study. Ann Med. 2023; 55(2):2239828. https://doi.org/10.1080/07853890.2023.2239828 PMID: 37498806
10. Anyama N, Adome RO. Community pharmaceutical care: an 8-month critical review of two pharmacies in Kampala. Afr Health Sci. 2003; 3(2):87–93. PMID: 12913800
11. Mayora C, Kitutu FE, Kandala NB, Ekirapa-Kiracho E, Peterson SS, Wamani H. Private retail drug shops: what they are, how they operate, and implications for health care delivery in rural Uganda. BMC Health Serv Res. 2018; 18(1):532. https://doi.org/10.1186/s12913-018-3343-z PMID: 29986729
12. Buss VH, Deeks LS, Shield A, Kosari S, Naunton M. Analytical quality and effectiveness of point-of-care testing in community pharmacies: A systematic literature review. Res Social Adm Pharm. 2019; 15(5):483–95. https://doi.org/10.1016/j.sapharm.2018.07.013 PMID: 30057328
13. Hamill MM, Onzia A, Parkes-Ratanshi RM, Kyambadde P, Mande E, Nakate V, et al. Antibiotic overuse, poor antimicrobial stewardship, and low specificity of syndromic case management in a cross section of men with urethral discharge syndrome in Kampala, Uganda. PLoS One. 2024; 19(3):e0290574. https://doi.org/10.1371/journal.pone.0290574 PMID: 38489281
14. Peeling RW, Holmes KK, Mabey D, Ronald A. Rapid tests for sexually transmitted infections (STIs): the way forward. Sex Transm Infect. 2006; 82 Suppl 5(Suppl 5):v1–6. https://doi.org/10.1136/sti.2006.024265 PMID: 17151023
15. Brabin L, Thomas G, Hopkins M, O'Brien K, Roberts SA. Delivery of chlamydia screening to young women requesting emergency hormonal contraception at pharmacies in Manchester, UK: a prospective study. BMC Womens Health. 2009; 9:7. https://doi.org/10.1186/1472-6874-9-7 PMID: 19323804

16. Figueira I, Teixeira I, Rodrigues AT, Gama A, Dias S. Point-of-care HIV and hepatitis screening in community pharmacies: a quantitative and qualitative study. Int J Clin Pharm. 2022; 44(5):1158–68. https://doi.org/10.1007/s11096-022-01444-1 PMID: 36098836

17. Mugo PM, Prins HA, Wahome EW, Mwashigadi GM, Thiong'o AN, Gichuru E, et al. Engaging young adult clients of community pharmacies for HIV screening in Coastal Kenya: a cross-sectional study. Sex Transm Infect. 2015; 91(4):257–9. https://doi.org/10.1136/sextrans-2014-051751 PMID: 25487430

18. Liambila W, Obare F, Keesbury J. Can private pharmacy providers offer comprehensive reproductive health services to users of emergency contraceptives? Evidence from Nairobi, Kenya. Patient Educ Couns. 2010; 81(3):368–73. https://doi.org/10.1016/j.pec.2010.09.001 PMID: 20934833

19. Lockhart A, Psioda M, Ting J, Campbell S, Mugo N, Kwatampora J, et al. Prospective Evaluation of Cervicovaginal Self- and Cervical Physician Collection for the Detection of Chlamydia trachomatis, Neisseria gonorrhoeae, Trichomonas vaginalis, and Mycoplasma genitalium Infections. Sex Transm Dis. 2018; 45(7):488–93. https://doi.org/10.1097/OLQ.0000000000000778 PMID: 29465667

20. Matsitse TB, Helberg E, Meyer JC, Godman B, Massele A, Schellack N. Compliance with the primary health care treatment guidelines and the essential medicines list in the management of sexually transmitted infections in correctional centres in South Africa: findings and implications. Expert Rev Anti Infect Ther. 2017; 15(10):963–72. https://doi.org/10.1080/14787210.2017.1382354 PMID: 28922959

21. Uganda Revenue Authority. Community Pharmacy and Drug Shops. https://uragoug/en/pharmacy-and-drug-shops/. 2024.

22. Kajubi P, Parkes-Ratanshi R, Twimukye A, Bwanika Naggirinya A, Nabaggala MS, Kiragga A, et al. Perceptions and Attitudes Toward an Interactive Voice Response Tool (Call for Life Uganda) Providing Adherence Support and Health Information to HIV-Positive Ugandans: Qualitative Study. JMIR Form Res. 2022; 6(12):e36829. https://doi.org/10.2196/36829 PMID: 36472904

23. Centers for Disease Control and Prevention. Sexually Transmitted Infections Guidelines. https://wwwcdcgov/std/treatment-guidelines/latent-syphilishtm. 2021.

24. Amesty S, Crawford ND, Nandi V, Perez-Figueroa R, Rivera A, Sutton M, et al. Evaluation of Pharmacy-Based HIV Testing in a High-Risk New York City Community. AIDS Patient Care STDS. 2015; 29 (8):437–44. https://doi.org/10.1089/apc.2015.0017 PMID: 26217930

25. Fu L, Tian T, Wang B, Lu Z, Bian J, Zhang W, et al. Global, regional, and national burden of HIV and other sexually transmitted infections in older adults aged 60–89 years from 1990 to 2019: results from the Global Burden of Disease Study 2019. Lancet Healthy Longev. 2024; 5(1):e17–e30. https://doi.org/10.1016/S2666-7568(23)00214-3 PMID: 38183996

26. https://phia.icap.columbia.edu/countries/uganda/. Summary Sheet: Uganda Population-Based HIV Impact Assessment 2020–2021. 2022.

27. Kiragga AN, Bwanika JM, Kyenkya J, Banturaki G, Kigozi J, Musinguzi D, et al. Point-of-care testing for HIV and sexually transmitted infections reveals risky behavior among men at gambling centers in Uganda". Int J STD AIDS. 2021; 32(10):903–10. https://doi.org/10.1177/09564624211000973 PMID: 33890852

28. Peters RPH, McIntyre JA, Garrett N, Brink AJ, Celum CL, Bekker LG. Doxycycline post-exposure prophylaxis for sexually transmitted infections in South Africa. South Afr J HIV Med. 2023; 24(1):1510. https://doi.org/10.4102/sajhivmed.v24i1.1510 PMID: 37795430

29. Grabowski MK, Mpagazi J, Kiboneka S, Ssekubugu R, Kereba JB, Nakayijja A, et al. The HIV and sexually transmitted infection syndemic following mass scale-up of combination HIV interventions in two communities in southern Uganda: a population-based cross-sectional study. Lancet Glob Health. 2022; 10(12):e1825–e34. https://doi.org/10.1016/S2214-109X(22)00424-7 PMID: 36400088

30. AIDS Control Programme of Uganda. Community Retail Pharmacy Drug Distribution Point (CRPDDP) Implementation Toolkit. https://pdfusaidgov/pdf_docs/PA00TBZ6pdf. 2021;1.
