# Supplementary material for: Client, health care provider, and policymaker perspectives on point-of-care/near-point-of-care testing for sexually transmitted infections in community pharmacies in Uganda
Source: PLoS One. 2026 Oct 15;21(10):e0347364. doi: 10.1371/journal.pone.0347364 (PMC13671497; doi:10.1371/journal.pone.0347364)
Supplement: Supplementary file 2 [file pone.0347364.s002.pdf]

Community Pharmacies for Assessing STI Using Point of Care Diagnostics Study (COPHAS)

Principal Investigators: Dr. Agnes Kiragga and Dr. Rosalind Parkes-Ratanshi

In-depth Interview Guide for Pharmacy Clients
Version 2.0 dated 22nd February 2022

Introduction

Hello **name,** you have been selected to participate in an on-going study titled "Community Pharmacy f Assessing STIs using Point of Care Diagnostics study (COPHAS)". We want to know your knowledge ar attitudes towards STIs, Point of Care Diagnostic Test Kits (POC), self-collected sample specimens ar delivery of test results at community pharmacies.

We shall continue making deliberate efforts to respect your privacy and confidentiality and guarantee yo information will only be accessed for study purposes. We seek your consent to record during the intervie We appreciate your commitment to making this study a success.

Respondent Demographics:

Interview ID: ……………… Sex:……….. Age:……….. Occupation: ……………..

Questions:

Background check questions

1.To start our discussion, I would like to understand your previous knowledge and views about POC te kits/self-sample collection at community pharmacies.

Knowledge

1. **Before** you became part of this study, had you ever heard of the term POC test kits/self-sample collection *(If YES, Probe for their previous understanding; previous experience in using POC tes previous opinion about it; If NO, when did you first hear about it).*

Practice

2. **Recently,** you have been part of this study:
   - a) What is your opinion about the study? *(Probe for what they liked; disliked and why; whether they thi the study was important or unimportant and why).*
   - b) What effect did participation in this study have on you? *(Probe for what they learnt; what they still not know about POC test kits).*
   - c) If you used POC tests kits, what is your opinion about them? (*Probe for what they like and benefi dislike and challenges; cost effectiveness of the tests; whether they shared tests results freely with pharmac and why or why not; whether they trusted the tests; what deters others from using POC tests pharmacies*).
   - d) What is your perspective as regards improving POC test kits/self-sample collection? *(Probe f their suggestions/recommendations; what pharmacies can do better, what the Ministry of Health can*

APPROVED
EFFECTIVE 25 MAY 2023 EXPIRY 09 DEC 2023
JOINT CLINICAL RESEARCH CENTRE REC

better; how the kits can be improved).

Communication

3. Through the period of study engagement or even shortly after:
   a) Did you ever discuss any POC/self-sample collection issues? *(First probe for whom they discuss with? Was it with spouse/partner? Friends? Colleagues? Community? what was discussed; probe f attitudes of those with whom they discussed).*

   b) Did you/your partner ever use any POC test kit during or after joining the study? *(Probe for wh they tested for; whether they tested for STI? HIV? Were the tests done before seeking treatment; did t partner like and/or dislike it and why).*

Impact assessment

4. Basing on the information shared about this study:

   a) Do you think that with the introduction of POC test kits at community pharmacies, perso seeking treatment would embrace testing first before seeking for treatment?

   b) Which other information would you be interested in receiving at the community pharmacy oth than the treatment options?

   c) What would be a better way of organizing testing and treatment services at communi pharmacies?

   d) Would you recommend POC test kits to a friend? If yes why and if no, why not?

5. Partner notification

   a) Did you share your test results with your spouse/partner? (*If YES, probe for how they did th spouse's reaction; If NO, why not?*).

   b) Did you receive notifications to share your test results with your partner/spouse? (If *YES, probe f opinions about it; whether it worked; whether they would recommend it; how it can be improved; If N explain why you did not sign up for it).*

   c) How was your experience of being diagnosed for an STI in a pharmacy? *(Probe for time spent study procedures; results receipt; follow-up; treatment given as compared to the health centers)*

Might you have any questions, comments or recommendations as regards POC/Self-sample collection issu at community pharmacies?

APPROVED
EFFECTIVE ★ 25 MAY 2023
EXPIRY 09 DEC 2023 ★
JOINT CLINICAL RESEARCH CENTRE REC

Thank you so much for invaluable contribution to this study.

END

APPROVED
EFFECTIVE EXPIRY
★ 2 5 MAY 2023 0 9 DEC 2023 ★
JOINT CLINICAL RESEARCH CENTRE
REC
