# Supplementary material for: Client, health care provider, and policymaker perspectives on point-of-care/near-point-of-care testing for sexually transmitted infections in community pharmacies in Uganda
Source: PLoS One. 2026 Oct 15;21(10):e0347364. doi: 10.1371/journal.pone.0347364 (PMC13671497; doi:10.1371/journal.pone.0347364)
Supplement: Supplementary file 3 [file pone.0347364.s003.pdf]

Community Pharmacies for Assessing STI Using Point of Care Diagnostics Study (COPHAS)

Principal Investigators: Dr. Agnes Kiragga and Dr. Rosalind Parkes-Ratanshi

Key Informant Interview Guide for Pharmacists/Dispensers and Pharmacy Proprietors

Version 2.0: Dated 22nd February 2022

Introduction:

Hello **name,** you have been selected to participate in an on-going study titled "Community Pharmacy f Assessing STIs using Point of Care Diagnostics study (COPHAS)". We want to know your knowledge ar attitudes towards STIs, PrEP, Point of Care Diagnostic Test Kits (POC), self-collected sample specimens ar delivery of test results at community pharmacies.

We shall continue making deliberate efforts to respect your privacy and confidentiality and guarantee yo information will only be accessed for study purposes. We seek your consent to record during the intervie We appreciate your commitment to making this study a success.

Respondent Demographics:

Interview ID: ……………… Sex:……….. Occupation: …………….. Pharmacy location………..

Questions:

1.To start with, I would like to understand your knowledge and views about PrEP:

a) What do you know about PrEP? (*Probe for what they know and then for knowledge of categories of persons that need PrEP?)*

b) Have you ever prescribed PrEP to anyone from your pharmacy? (*If YES, how was the experience? Enablers and benefits; challenges and obstacles; patients' attitudes towards PrEP If NO probe for why not)*

c) Would you prescribe PrEP to a high-risk person at your pharmacy? Explain.

d) What recommendations would you give to improve the prescription of PrEP at community pharmacies?

2. Recently, you have been part of this study:

e) What is your opinion about the study? *(Probe for what they liked; disliked and why; whether they think th study was important or unimportant and why).*

f) What effect did participation in this study have on you? *(Probe for what they learnt; what they still do n know about POC test kits).*

g) If you used POC tests kits, what is your opinion about them? (*Probe for what they like and benefits; dislike and challenges; whether patients were receptive in using them and why or why not; whether patients shared tests results freely with pharmacist and why or why not; whether pharmacists trusted the tests; what deters patients from using POC tests at pharmacies; what can be done to encourage them*).

APPROVED
EFFECTIVE EXPIRY
★ 25 MAY 2023 09 DEC 2023 ★
JOINT CLINICAL RESEARCH CENTRE
REC

h) Basing on the information shared about this study do you think the introduction of POC test kits at community pharmacies would influence testing first before seeking for treatment? *(Explain; probe for cost effectiveness for patients and the pharmacy)*

i) What is your perspective as regards improving POC test kits/self-sample collection? *(Probe for their suggestions/recommendations; what pharmacies can do better; what the Ministry of Health can do better; how the kits can be improved).*

3. a) As far as you know, did patients share test results with their spouses/partners? (*If YES, probe for how they did this; spouse reactions; If NO, why not?*)

b) Did you support patients towards sharing test results with spouses/partners? (*If YES, how did you do it? If NOT, why not?)*

c) Did you send notifications for patients to share test results with partners/spouses? (If *YES, probe for whether it worked and why or why not; If NO, explain why you did)*

d) What is your opinion as regards patients' notifications (*Probe for benefits; challenges; whether they would recommend their use; how the service can be improved)*

4. What do you know about STI and HIV treatment guidelines and principles of antimicrobial stewardship of pharmacy staff? *(Probe for their opinions about these guidelines; benefits; challenges/stumbling block recommendations to improve them*).

5. What is the impact of these regulations on your business? (*Probe for their opinions on if the NDA regulation work; If not why; stumbling blocks and recommendations to ensure they are on board with the AMR stewardship)*

6. Might you have any questions, comments or recommendations as regards POC/Self-sample collection issues at community pharmacies?

Thank you so much for invaluable contribution to this study.

END

APPROVED
EFFECTIVE EXPIRY
★ 2 5 MAY 2023 0 9 DEC 2023 ★
JOINT CLINICAL RESEARCH CENTRE
REC
