# Supplementary material for: Client, health care provider, and policymaker perspectives on point-of-care/near-point-of-care testing for sexually transmitted infections in community pharmacies in Uganda
Source: PLoS One. 2026 Oct 15;21(10):e0347364. doi: 10.1371/journal.pone.0347364 (PMC13671497; doi:10.1371/journal.pone.0347364)
Supplement: Supplementary file 4 [file pone.0347364.s004.PDF]

Codebook – Perspectives on POC STI Testing in Community Pharmacies (CPs) in Uganda, June 2022.

Theme	Sub-theme / Code	Description	Illustrative Quote
**1. Awareness of POC Testing was Low, Although Once Educated, Many Perceived Its Importance**	Low awareness of POC testing	Many clients were unaware of the existence of HIV/STI POC testing at community pharmacies, often discovering them incidentally.	"No, I had never heard about it..." — *IDI, Male, Symptomatic*
	Perceived quality and comprehensiveness of services	Participants valued the comprehensive nature of services—counselling, education, treatment, and follow-up—which supported positive health outcomes.	"…I felt scared a bit, but then since I received good counselling, I said let me enroll for treatment…" — *IDI, Male, Symptomatic*
	Health education encourages behavioural change	Health education during testing encouraged responsible sexual behaviour, such as monogamy and regular testing.	"...after this testing, I chose to stick to my officially introduced wife and I openly told her...let me settle down ..." — *IDI, Male, Asymptomatic*
**2. Affordability of POC Testing Increases Access and Diagnostic Certainty**	Affordability and access	Free or low-cost testing improved accessibility compared to conventional laboratory testing.	"Their services were good, and yet they were free..." — *IDI, Female, Asymptomatic*
	Diagnostic certainty and targeted treatment	POC testing enabled accurate diagnosis and targeted treatment, improving outcomes and reducing antimicrobial misuse.	"…In the context of antimicrobial resistance, this could go miles in reducing the time…" — *KII, MOH Consultant*
	Ease of partner notification	Testing encouraged clients to refer sexual partners for testing and treatment, reducing reinfection risks.	"I took it upon myself to inform my partner to get tested after me…." — *IDI, Male, Symptomatic*

	Convenience and accessibility of CPs	Pharmacies were seen as trusted, convenient, and well-positioned to reach communities in both rural and urban areas.	"…community pharmacies are very accessible…." — *KII, MOH Technical Advisor*
	Promptness of results	The short turnaround time for results enabled prompt treatment initiation.	"It saves a lot of time…" — *KII, Pharmacy Dispenser*
**3. Lack of Awareness, Privacy, and Accuracy Concerns Pose Barriers to POC Testing**	Limited awareness among clients and providers	Both clients and pharmacists lacked awareness of STI POC testing beyond HIV.	"There is a knowledge gap…." — *KII, Male, Pharmacist*
	Privacy and space limitations	Lack of private testing spaces discouraged participation and openness.	"…the pharmacy was in the open and people kept passing…" — *IDI, Female, Symptomatic*
	Difficulties with self-sampling	Participants reported discomfort or confusion during self-sample collection, affecting accuracy.	"At some point, I didn't know how deep I was to push the swab." — *IDI, Female, Symptomatic*
	Anxiety, stigma, and fear	Long waits and fear of HIV stigma discouraged testing.	"Some people are afraid to know their status..." — *KII, Male, Pharmacy Technician*
	Concerns over test accuracy and training	Doubts about reliability and insufficient pharmacist training raised concerns about false results.	"The challenge with POCs is sensitivity and specificity..." — *KII, Male, Consultant Medical Officer*
	Potential misuse and profit-driven motives	Pharmacies' financial motivations could lead to misuse or inappropriate treatment.	"It happens a lot in pharmacies... dispensers are on pressure..." — *IDI, Female, Asymptomatic*
**4. Recommendations to Improve POC Testing at CPs**	Increase awareness and reduce stigma	Participants recommended awareness campaigns and locally tailored communication to normalize STI testing.	"It would be good if things like flyers are put there..." — *IDI, Female, Symptomatic*
	Improve privacy and access	Suggested dedicated testing days and temporary private spaces (e.g., tents) to	"We get like a tent, that is placed on the side, so that the client and the health worker..." — *IDI, Female, Symptomatic*

		enhance confidentiality.	
	Enhance test acceptability	Advocated for less invasive sample types and translation of instructions into local languages.	“...let them even try to bring it in Luganda [local language] to make it easy for someone to read it.” — *KII, Female, Nurse/Dispenser*
	Broaden STI test coverage	Recommended POC kits that detect a wider range of STIs, not only HIV.	“STIs are very many and thus the point of care should work on a wide range of STIs, not mainly HIV.” — *KII, Male, STI Control Unit*
	Strengthen regulation, training, and guidelines	Recommended continuous training, supervision, and updated policies to minimize AMR and improve practice.	“We should really have this constant training to update our knowledge.” — *KII, Female, Pharmacist*
	Ensure supply chain sustainability	Ensuring consistent availability of test kits and medicines to sustain services.	“…the supply chain has to be guaranteed and sustainable.” — *KII, Pharmacist, Supply Chain Expert*
